# Supplementary material for: Mgl2+ cDC2s coordinate fungal allergic airway type 2, but not type 17, inflammation in mice
Source: Nat Commun. 2025 Jan 22;16:928. doi: 10.1038/s41467-024-55663-3 (PMC11754877; doi:10.1038/s41467-024-55663-3)
Supplement: Supplementary file 1 — Supplementary Information [file 41467_2024_55663_MOESM1_ESM.pdf]

Supplementary Table 1. List of qPCR primers

Gene	Accession No.	Fwd Primer	Rev Primer	Amplicon size (b.p.)
Hprt	NM_013556.2	TCCTCCTCAGACCGCTTTT	CCTGGTTCATCATCGCTAATC	90
Il4	NM_021283.2	GAGAGATCATCGGCATTTTGA	TCTGTGGTGTTCTTCGTTGC	100
Il5	NM_010558.1	ACATTGACCGCCAAAAAGAG	CACCATGGAGCAGCTCAG	136
Il10	NM_010548.2	CAGAGCCACATGCTCCTAGA	TGTCCAGCTGGTCCTTTGTT	79
Il13	NM_008355.3	CCTCTGACCCTTAAGGAGCTTAT	CGTTGCACAGGGGAGTCT	70
Il17	NM_010552.3	TGTGAAGGTCAACCTCAAAGTC	AGGGATATCTATCAGGGTCTTCATT	131
Ifng	NM_008337.4	GGAGGAACTGGCAAAAGGAT	TTCAAGACTTCAAAGAGTCTGAGG	85

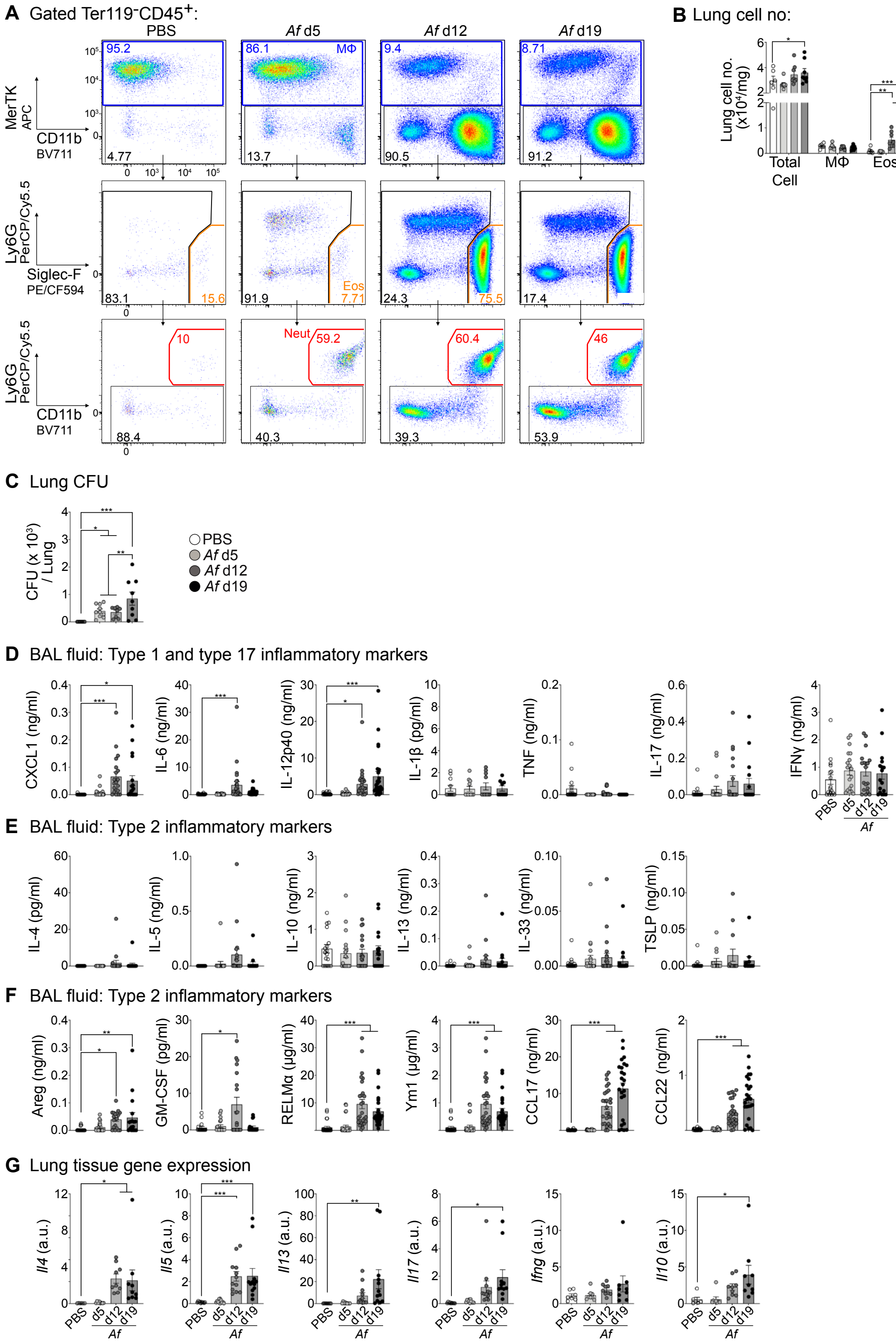

Supplementary Figure 1. Repeat exposure to fungal spores induces type 2 and type 17 mediators in lung airway and tissue. Mice were repeatedly exposed intranasally to PBS or *Af* spores (4 x 10^5 per dose) and tissues harvested the day after the third, sixth or ninth dose of spores (d5, d12 and d19, respectively). (**A**) Flow cytometry plots identifying macrophages (MΦs), eosinophils (Eos) and neutrophils (Neut) isolated from the BAL fluid of mice following exposure to three, six or nine doses of *Af* spores or PBS controls. (**B**) Graph displays the number of MΦs, Eos, Neut and monocytes (Mono) from the lung tissue. (**C**) Graph displays lung tissue fungal burden, as measured by colony forming units (CFU) of whole lung tissue, following repeat doses of PBS or *Af* spores. (**D - F**) ELISA of mediators in BAL fluid following repeat does of *Af* spores or PBS. (**G**) mRNA expression by quantitative PCR (qPCR) of lung tissue following repeat doses of *Af* spores or PBS. **B**, data from 2 independent experiments (n = 30 biologically independent animals). **C**, data from 2 independent experiments (n = 37 biologically independent animals). **D**, CXCL1 data from 4 independent experiments (n = 76 biologically independent animals), IL-6 & IL-12p40 data from 7 independent experiments (n = 104 biologically independent animals), IL-1β data from 3 independent experiments (n = 42 biologically independent animals), TNF, IL-17 & IFNγ data from 5 independent experiments (n = 72 biologically independent animals). **E**, IL-4, IL-5, IL-10 & IL-13 data

from 5 independent experiments (n = 72 biologically independent animals), IL-33 data from 6 independent experiments (n = 88 biologically independent animals), TSLP data from 4 independent experiments (n = 54 biologically independent animals). **F**, Areg & GM-CSF data from 5 independent experiments (n = 72 biologically independent animals), RELMα, Ym1, CCL17 & CCL22 data from 7 independent experiments (n = 104 biologically independent animals). **G**, *Il4*, *Ifng* & *Il10* data from 2 independent experiments (n = 33 biologically independent animals), *Il5, Il13* & *Il17* data from 3 independent experiments (n = 45 biologically independent animals). Data were fit to a linear mixed effect model, with experimental day as a random effect variable, and groups compared with a two-sided Tukey's multiple comparison test. *p < 0.05, **p < 0.01, ***p < 0.001. Data are presented as mean values ± SEM. Source data are provided as a Source Data File.

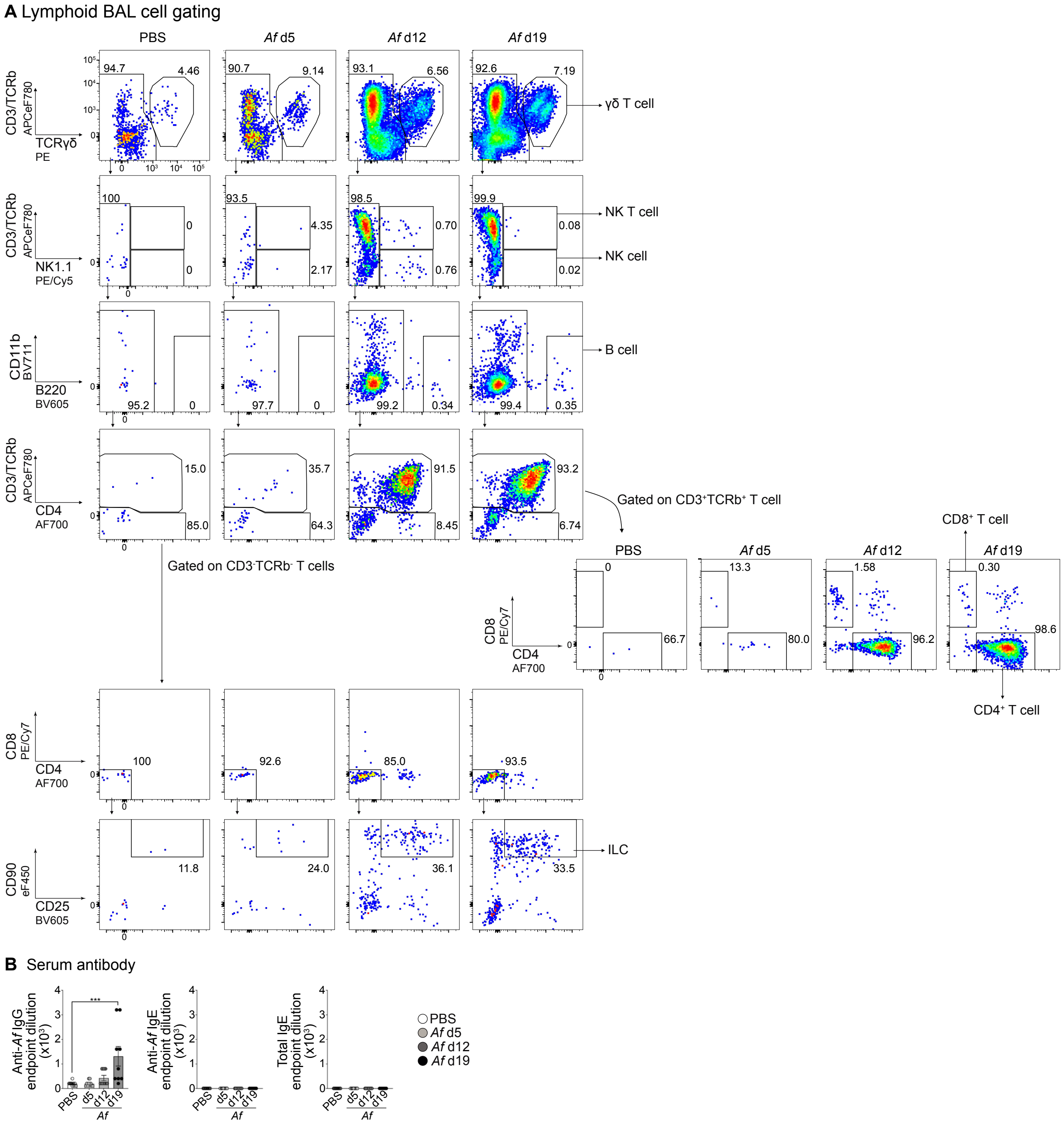

Supplementary Figure 2. Repeat exposure to fungal spores increases lymphoid cell populations in the airways, and fungal-specific antibody in serum. Mice were repeatedly exposed intranasally to PBS or *Af* spores (4 x 10^5 per dose) and tissues harvested the day after the third, sixth or ninth dose of spores (d5, d12 and d19, respectively). (**A**) Representative flow cytometry plots showing gating for cells from BAL fluid to identify (following gating on live, $CD45^+MerTK^-SiglecF^-Ly6G^-$ single cells) TCRγδ T cells, NK cells, NK T cells, B cells, $CD4^+$ T cells, $CD8^+$ T cells and ILC populations. (**B**) Graphs show ELISA of serum antibodies following repeat doses of *Af* spores or PBS. Data from 2 independent experiments (n = 37 biologically independent animals). Data were fit to a linear mixed effect model, with experimental day as a random effect variable, and groups compared with a two-sided Tukey's multiple comparison test. ***$p < 0.001$. Data are presented as mean values ± SEM. Source data are provided as a Source Data File.

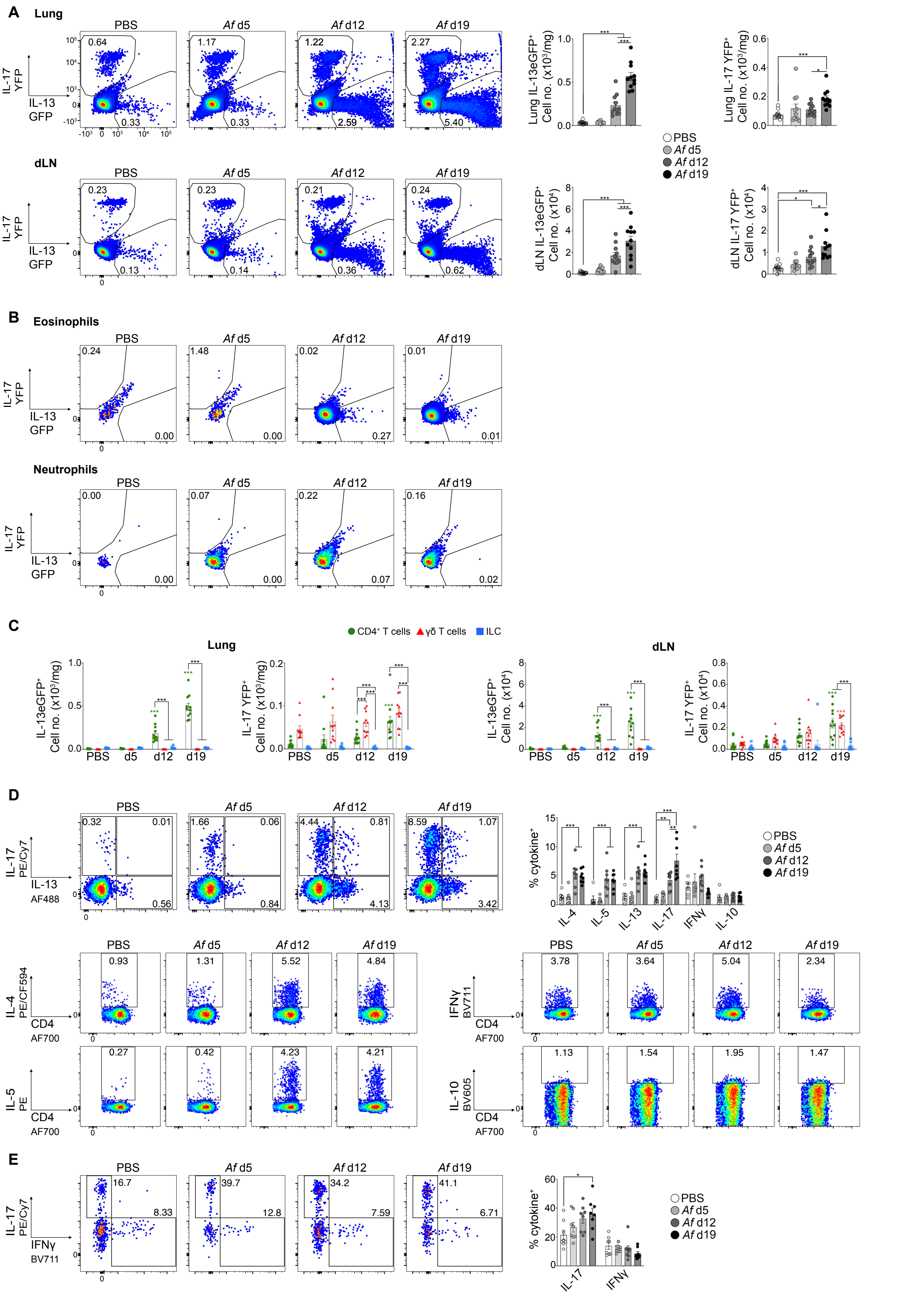

Supplementary Figure 3. Repeat exposure to fungal spores increases type 2 and type 17 cytokine expressing cells in lung tissue and draining LN. (**A**) Representative flow cytometry plots and graphs identify the proportion and number of *Il13* and *Il17* expressing cells isolated from the lung tissue and dLN of *Il13*^{eGFP}*Il17*^{Cre}*ROSA*^{eYFP} mice exposed intranasally to varying doses of *Af* spores (4 x 10^5 per dose). (**B**) Representative flow cytometry plots show eosinophil and neutrophil

expression of *Il13* and *Il17* expressing cells isolated from the BAL fluid of *Il13*^{eGFP}*Il17*^{Cre}*ROSA*^{eYFP} mice exposed to varying doses of *Af* spores. (**C**) Graphs show the number of *Il13* and *Il17* cells CD4$^+$ T cells (green), γδ T cells (red), and ILCs (blue) that were detected in the lung and dLN of *Il13*^{eGFP}*Il17*^{Cre}*ROSA*^{eYFP} mice exposed to varying doses of *Af* spores. (**D**) Representative flow cytometry plots identify IL-4$^+$, IL-5$^+$, IL-13$^+$, IL-10$^+$, IL-17$^+$ and IFNγ$^+$ populations via intracellular cytokine staining of lung CD4$^+$ T cells post stimulation with PMA/ionomycin. Graphs show the proportion of CD4$^+$ T cells expressing type 1, type 2 and type 17 cytokine. (**E**) Representative flow cytometry plots identify IFNγ$^+$ and IL-17$^+$ populations via intracellular cytokine staining of γδ T cells post stimulation with PMA/ionomycin. **A** & **C**, data from 3 independent experiments (n = 44 biologically independent animals). **D**, data from 2 independent experiments (n = 30 biologically independent animals). **E**, data from 2 independent experiments (n = 32 biologically independent animals). Data were fit to a linear mixed effect model, with experimental day as a random effect variable, and groups compared with a two-sided Tukey's multiple comparison test. *$p < 0.05$, **$p < 0.01$, ***$p < 0.001$. Data are presented as mean values ± SEM. Source data are provided as a Source Data File.

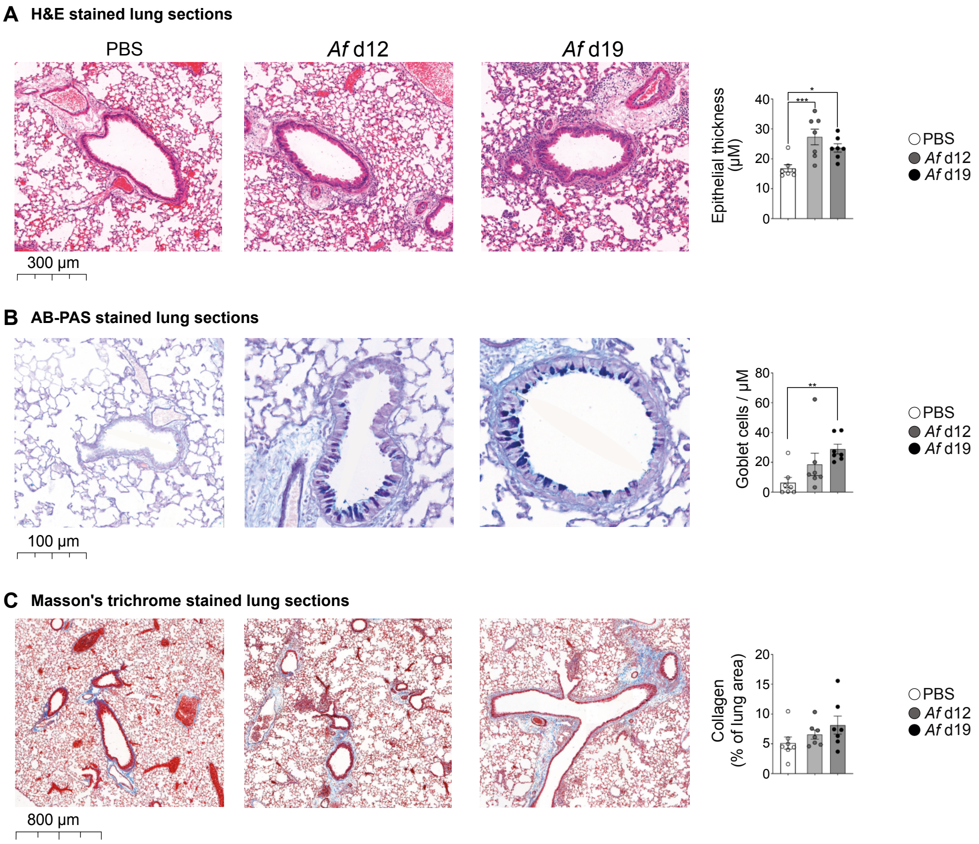

Supplementary Figure 4. **Repeat exposure to fungal spores triggers allergic airway inflammation in the lung.** Representative images show lung tissue sections from mice exposed intranasally to PBS or varying doses of *Af* spores (4 x 10^5 per dose), stained with (**A**) H&E, (**B**) AB-PAS and (**C**) Masson's Trichrome. **A**, **B** & **C**, data from 2 independent experiments (n = 21 biologically independent animals). Data were fit to a linear mixed effect model, with experimental day as a random effect variable, and groups compared with a two-sided Tukey's multiple comparison test. *$p < 0.05$, **$p < 0.01$, ***$p < 0.001$. Data are presented as mean values ± SEM. Source data are provided as a Source Data File.

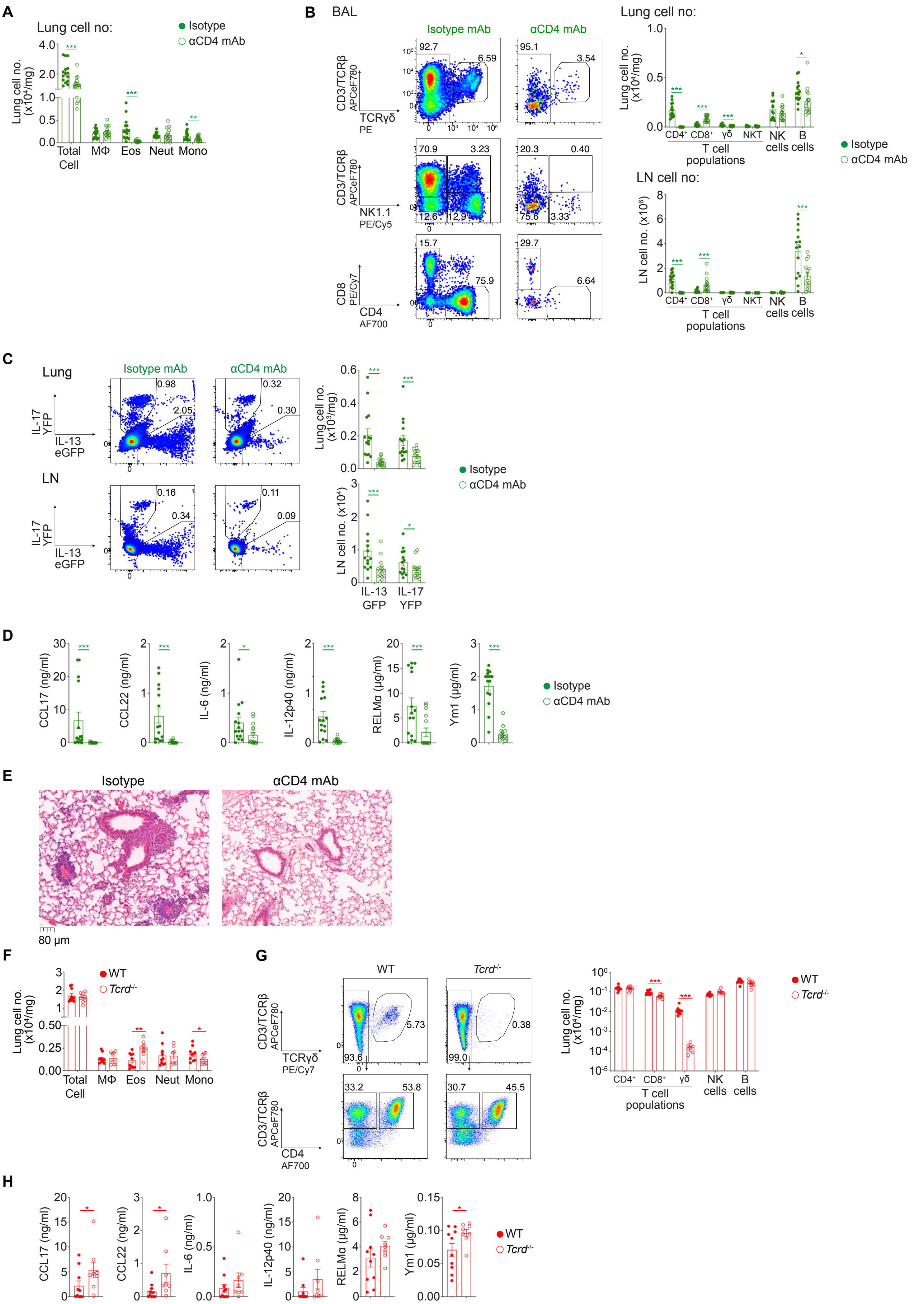

Supplementary Figure 5. Type 2 and type 17 fungal allergic airway inflammation is dependent on CD4⁺ T cells. (**A - D**) *Il13*^{eGFP}*Il17*^{Cre}*ROSA*^{eYFP} mice were treated with anti-CD4 mAb or isotype control mAb on d6 after three doses of *Af* spores (4 x 10^5 per dose) and subsequently exposed to three further doses of spores. BAL fluid, lung and dLN tissue were harvested 24h after

the sixth dose. (**A**) Graph show the number of macrophages (MΦs), eosinophils (Eos) neutrophils (Neut), and monocytes (Mono) from the lung tissue. (**B**) Flow cytometry plots identifying different lymphocyte populations alongside graphs showing the cell numbers isolated from the BAL fluid of mice. Graphs show the number of lymphocyte populations from the lung tissue and dLN. (**C**) Representative flow cytometry plots and graphs identify the proportion and number of *Il13* and *Il17* expressing cells isolated from the lung and dLN. (**D**) ELISA of mediators detected from the BAL fluid. (**E**) Representative images of lung tissue stained with H&E. **(A – D)**, data pooled from three independent experiments (n = 15 mice per group). (**E - G**) WT or $Tcrd^{-/-}$ mice were exposed intranasally to six doses of *Af* spores (4 x 10^5 per dose), BAL fluid and lung tissue were harvested 24h after the final dose. (**E**) Graph show the number of MΦ, Eos, Neut and monocytes (Mono) from the lung tissue. (**F**) Flow cytometry plots identifying γδ T cells and $CD4^+$ T cell populations alongside graphs showing the cell numbers isolated from the BAL fluid of mice. Graphs show the number of lymphocyte populations from the lung tissue. (**G**) ELISA of mediators detected from the BAL fluid. **A, B (lung), C (lung) & D**, data from 3 independent experiments (n = 30 biologically independent animals). **B (LN) & C (LN)**, data from 3 independent experiments (n = 29 biologically independent animals). **F**, **G** & **H** data from 2 independent experiments (n = 18 biologically independent animals). Data were fit to a linear mixed effect model, with experimental day as a random effect variable, and groups compared with a two-sided Tukey's multiple comparison test. *p < 0.05, **p < 0.01, ***p < 0.001. Data are presented as mean values ± SEM. Source data are provided as a Source Data File.

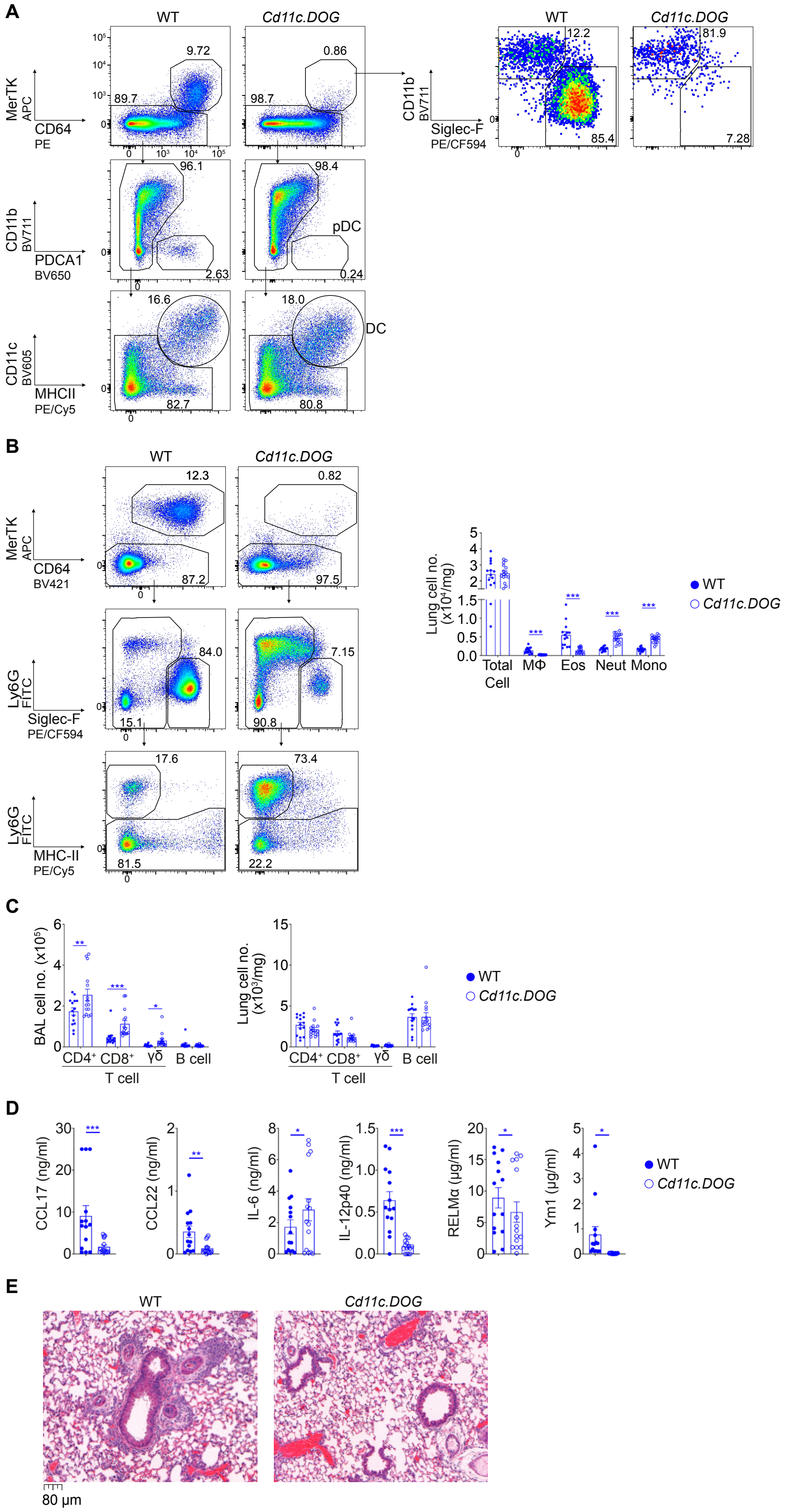

Supplementary Figure 6. CD11c-expressing cell depletion disrupts fungal allergic airway inflammation. WT or *Cd11c.DOG* mice were treated with diphtheria toxin (DTx) on d6 after three doses of *Af* spores (4 x 10^5 per dose) and subsequently exposed to three further doses of spores. BAL fluid and lung tissue were harvested 24h after the sixth dose. (**A**) Representative flow cytometry plots show the impact of CD11c$^+$ depletion on myeloid populations in the lung tissue. (**B**) Representative flow cytometry plots of shows the identification of different populations in the BAL fluid. Graph show the number of macrophages (MΦs), eosinophils (Eos) neutrophils (Neut), and monocytes (Mono) from the lung tissue. (**C**) Graphs show the number of lymphocyte populations

from the BAL fluid and lung tissue. (**D**) ELISA of mediators detected from the BAL fluid. (**E**) Representative images of lung tissue stained with H&E. **B**, **C** & **D**, data from 3 independent experiments (n = 29 biologically independent animals). Data were fit to a linear mixed effect model, with experimental day as a random effect variable, and groups compared with a two-sided Tukey's multiple comparison test. *p < 0.05, **p < 0.01, ***p < 0.001. Data are presented as mean values ± SEM. Source data are provided as a Source Data File.

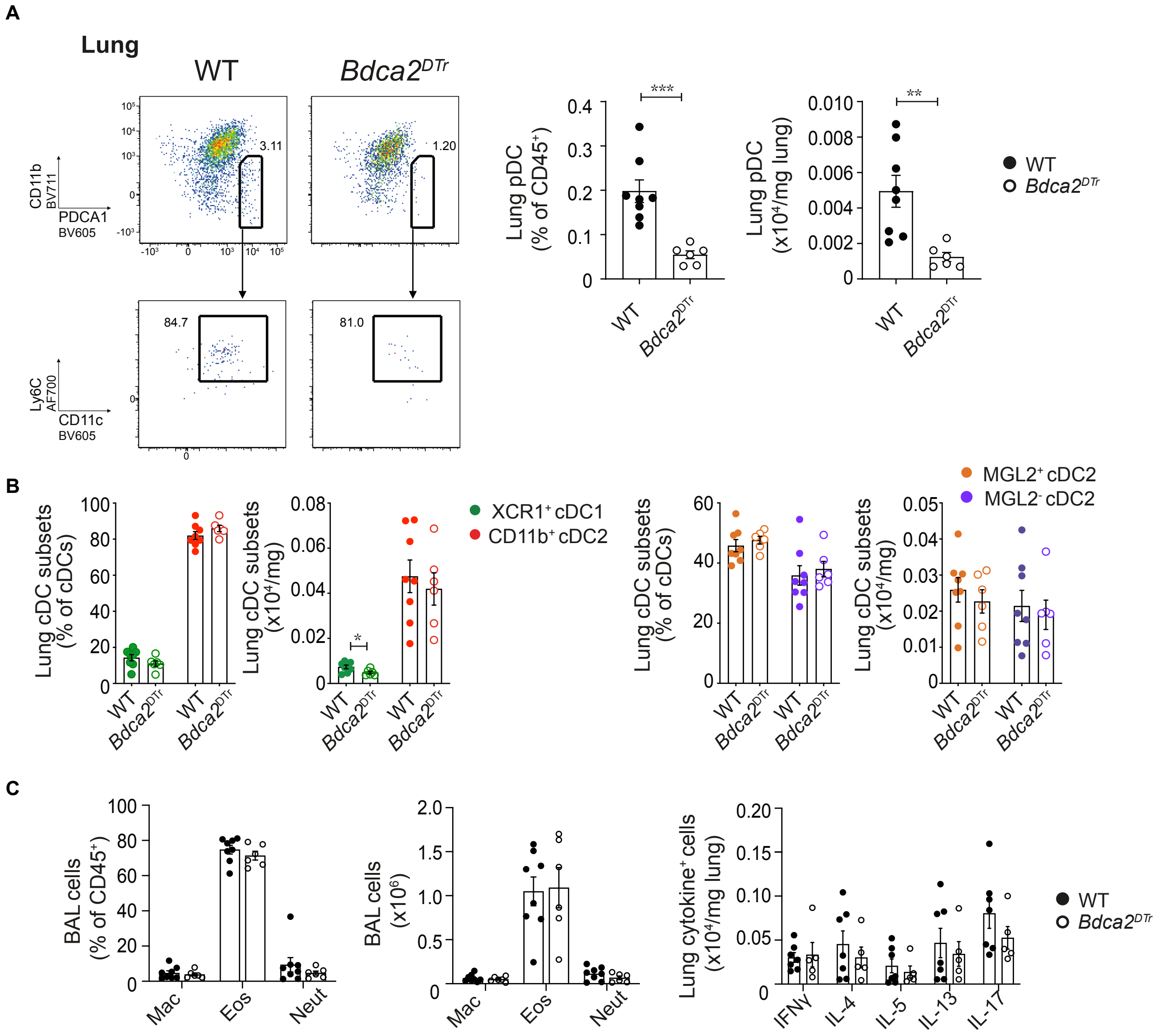

Supplementary Figure 7. Type 2 and type 17 fungal allergic airway inflammation is not dependent on pDCs. (**A**) WT or *Bdca2*^{DTR} mice were treated with diphtheria toxin (DTx) on d12, after six doses of *Af* spores (4 x 10^5 per dose) and subsequently exposed to three further doses of spores. BAL fluid and lung tissue were harvested 24h after the ninth dose. Flow cytometry plots identify pDC populations alongside graphs that display cell numbers that were detected in the lung tissue. (**B**) Graphs display the number and percentage of DC subsets that were detected in the lung tissue. (**C**) Graphs show percentage and number of macrophages (MΦs), eosinophils (Eos), neutrophil (Neut) from the BAL fluid. Graphs show the number of type 2 and type 17 cytokine lung CD4$^+$ T cells post stimulation with PMA/ionomycin. **A - C (BAL)**, data from 2 independent experiments (n = 14 biologically independent animals). **C (Lung cytokine)**, from 2 independent experiments (n = 12 biologically independent animals). Data were fit to a linear mixed effect model, with experimental day as a random effect variable, and groups compared with a two-sided Tukey's multiple comparison test. *$p < 0.05$, **$p < 0.01$, ***$p < 0.001$. Data are presented as mean values ± SEM. Source data are provided as a Source Data File.

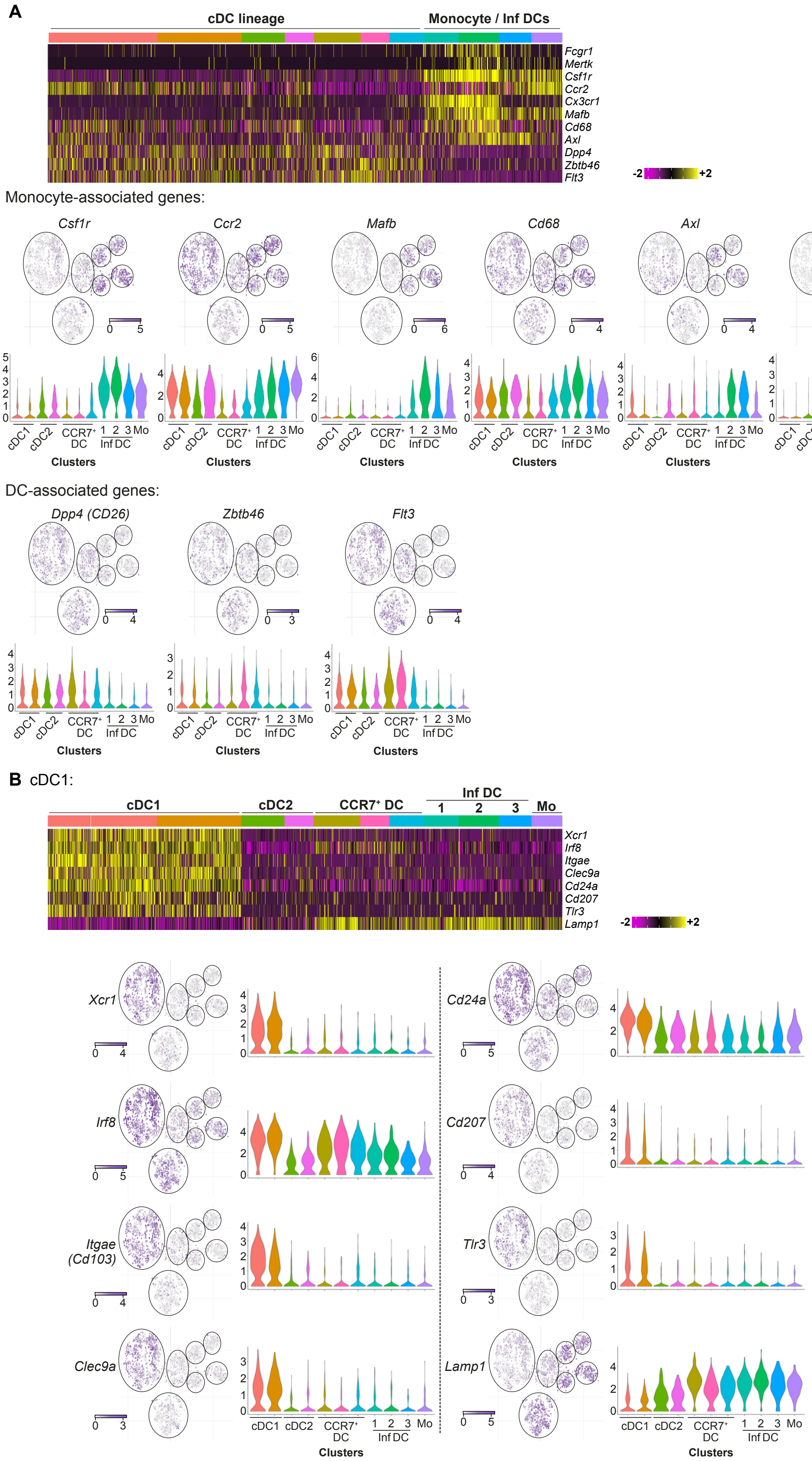

Supplementary Figure 8. Single cell RNA-sequencing of pulmonary DCs suggests distinct clusters of different DC lineages during fungal allergic airway inflammation. DCs were isolated from the lung tissue of naïve mice or mice that had been repeatedly exposed to *Af* spores (4 x 10^5 per dose, intranasally), harvested 24h after the third or sixth dose (*Af* d5 and *Af* d12, respectively). Single cell libraries were generated from these populations using the 10x chromium platform and sequenced (Illumina Hi-seq). 11 clusters were identified by dynamic tree cluster method and were refined to 7 populations using highly expressed genes (populations indicated on

heatmaps, tSNE and violin plots). (**A**) Heat map, tSNE and violin plots shows scaled expression of 11 genes associated with cDC versus monocyte/inflammatory DC development. (**B**) Heat map (scaled gene expression), tSNE and violin plots show expression of the top 8 genes that define the cDC1 cluster.

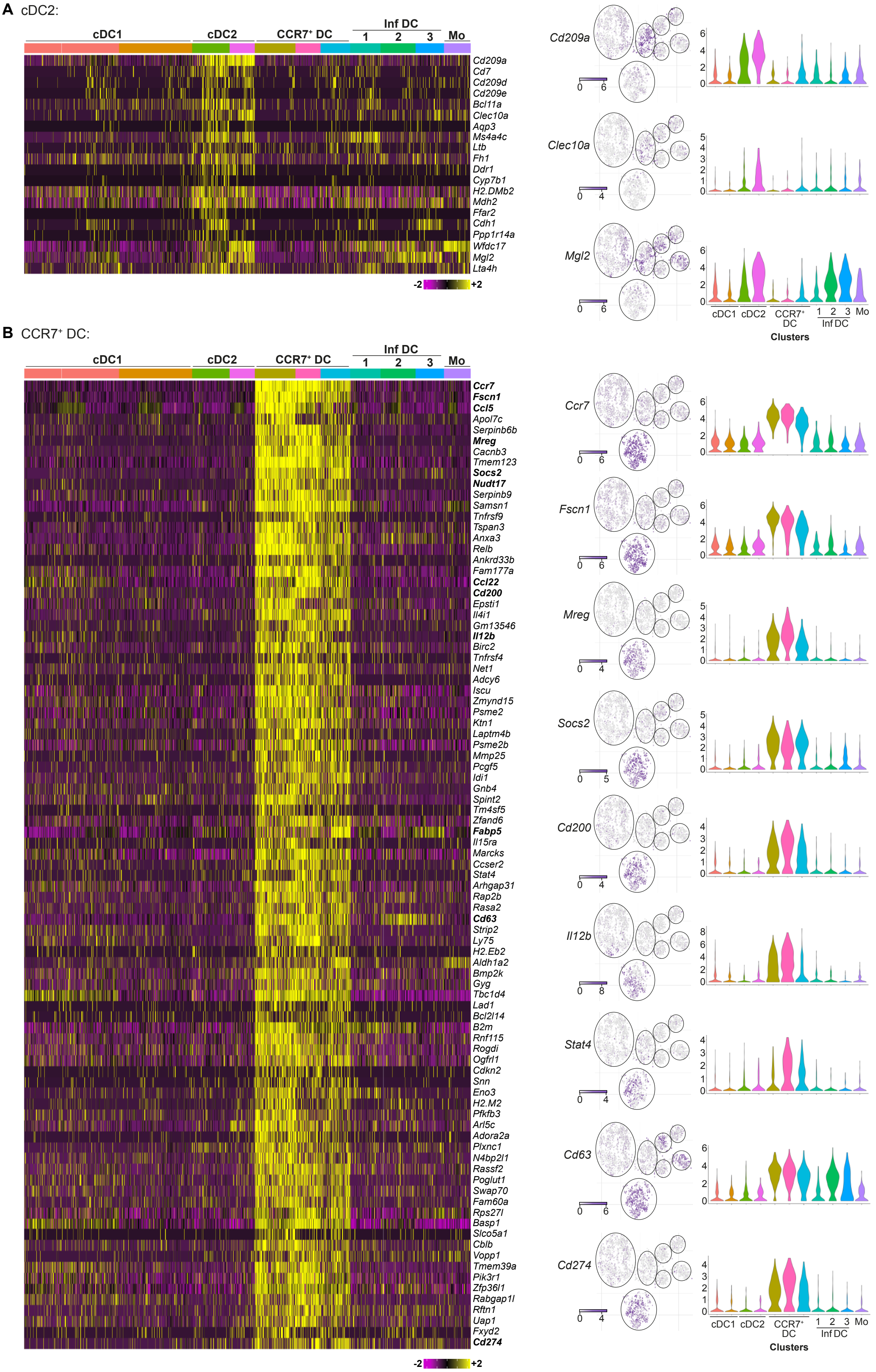

Supplementary Figure 9. Single cell RNA-sequencing of pulmonary DCs reveals cDC2 and

CCR7⁺ DC clusters during fungal allergic airway inflammation. DCs were isolated from the lung tissue of naïve mice or mice that had been repeatedly exposed to *Af* spores (4 x 10^5 per dose, intranasally), harvested 24h after the third or sixth dose (*Af* d5 and *Af* d12, respectively). Single cell libraries were generated from these populations using the 10x chromium platform and sequenced (Illumina Hi-seq). 11 clusters were identified by dynamic tree cluster method and were refined to 7 populations using highly expressed genes (populations indicated on heatmaps, tSNE and violin plots). (**A**) Heat map (20 genes) with selected tSNE and violin plots (3 genes) shows scaled expression of genes show cluster defining genes within the cDC2 cluster. (**B**) Heat map (89 genes) with selected tSNE and violin plots (9 genes) shows scaled expression of genes that define the CCR7⁺ DC cluster.

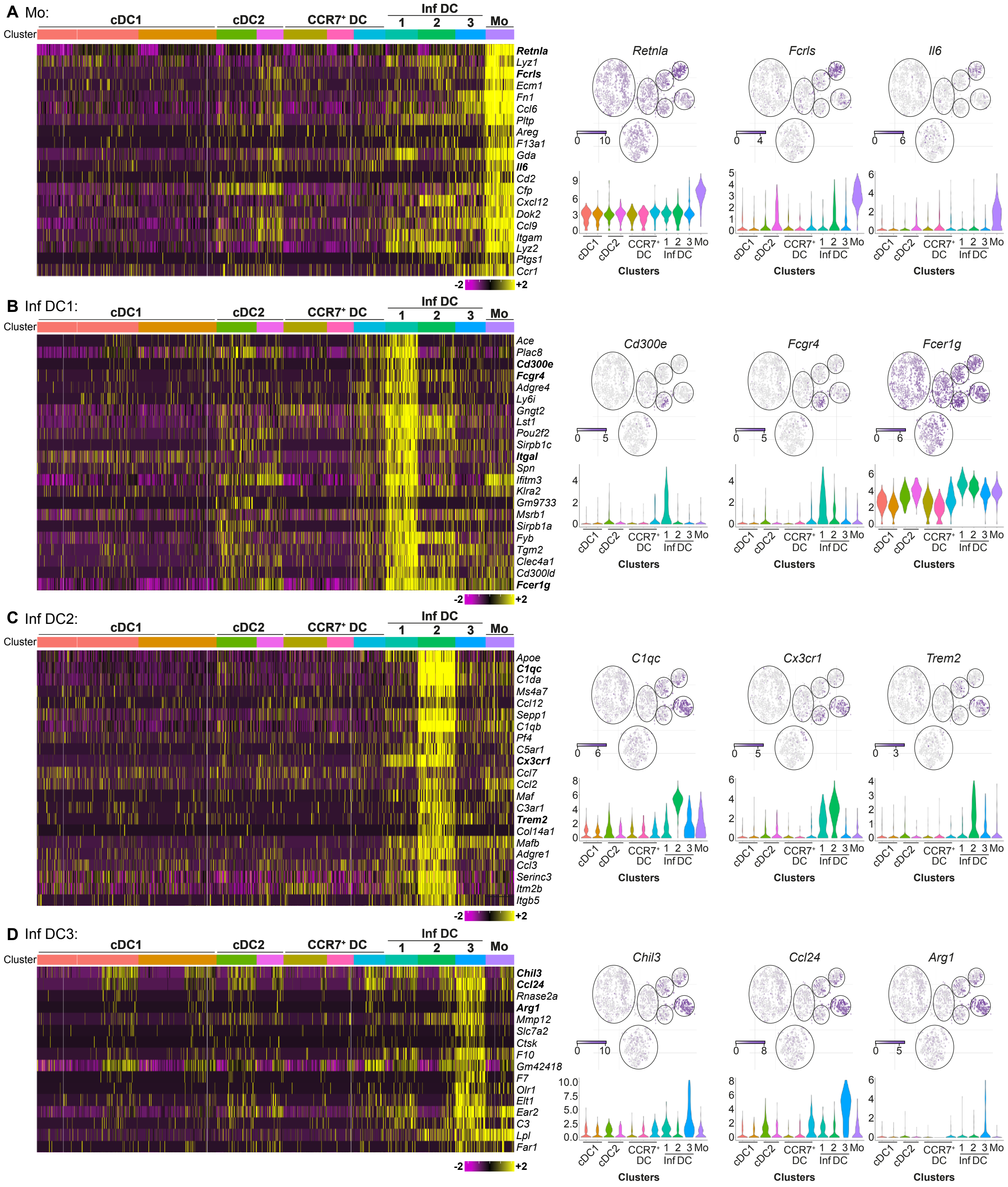

Supplementary Figure 10. Single cell RNA-sequencing of pulmonary DCs reveals inflammatory DC clusters during fungal allergic airway inflammation. DCs were isolated from the lung tissue of naïve mice or mice that had been repeatedly exposed to *Af* spores (4 x 10^5 per dose, intranasally), harvested 24h after the third or sixth dose (*Af* d5 and *Af* d12, respectively). Single cell libraries were generated from these populations using the 10x chromium platform and sequenced (Illumina Hi-seq). 11 clusters were identified by dynamic tree cluster method and were refined to 7 populations using highly expressed genes (populations indicated on heatmaps, tSNE and violin plots). (**A**) Heat map (20 genes) with selected tSNE and violin plots (3 genes) shows scaled expression of genes show cluster defining genes within the monocyte (Mo) cluster. (**B**) Heat map (22 genes) with selected tSNE and violin plots (3 genes) shows scaled expression of genes that define the Inf DC1 cluster. (**C**) Heat map (22 genes) with selected tSNE and violin plots (3 genes) shows scaled expression of genes that define the Inf DC2 cluster. (**D**) Heat map (15 genes) with selected tSNE and violin plots (3 genes) shows scaled expression of genes that define the Inf DC2 cluster.

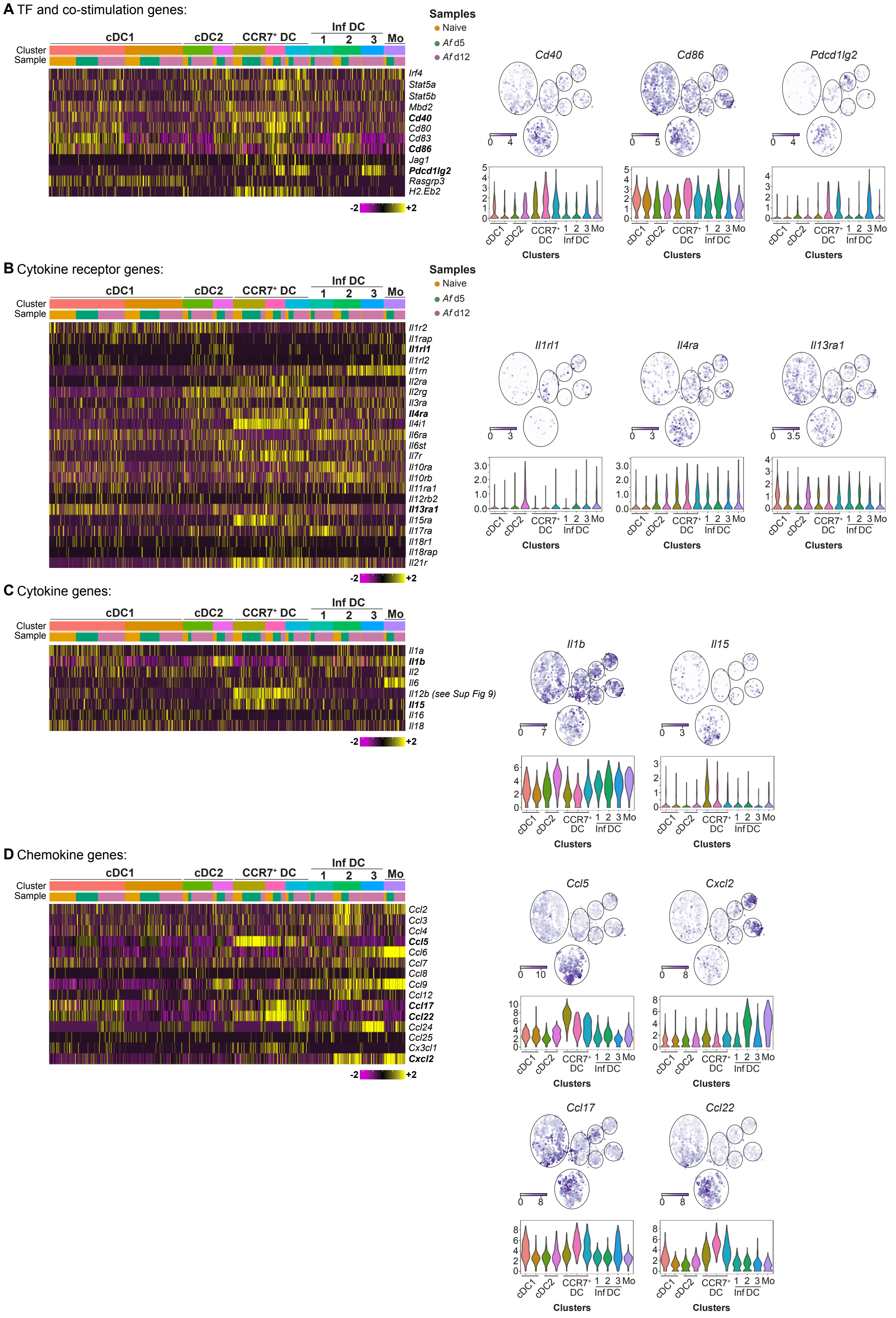

Supplementary Figure 11. Fungal allergic airway inflammation does not clearly alter expression of genes associated with DC function, as measured by single cell RNA-seq

sequencing. DCs were isolated from the lung tissue of naïve mice or mice that had been repeatedly exposed to *Af* spores (4 x 10^5 per dose, intranasally), harvested 24h after the third or sixth dose (*Af* d5 and *Af* d12, respectively). Single cell libraries were generated from these populations using the 10x chromium platform and sequenced (Illumina Hi-seq). 11 clusters were identified by dynamic tree cluster method and were refined to 7 populations using highly expressed genes (populations indicated on heatmaps, tSNE and violin plots). Heat maps (scaled gene expression) of selected genes associated with (**A**) DC development and activation, (**B**) cytokine receptors, (**C**) cytokines and (**D**) chemokines across the identified clusters. Within each cluster, samples of origin are shown from the lung tissue of naïve mice or mice repeatedly exposed *Af* spores, harvested 24h after the third and sixth dose (*Af* d5 and *Af* d12, respectively). For each heatmap, tSNE and violin plots show expression of 3 selected genes within each cluster.

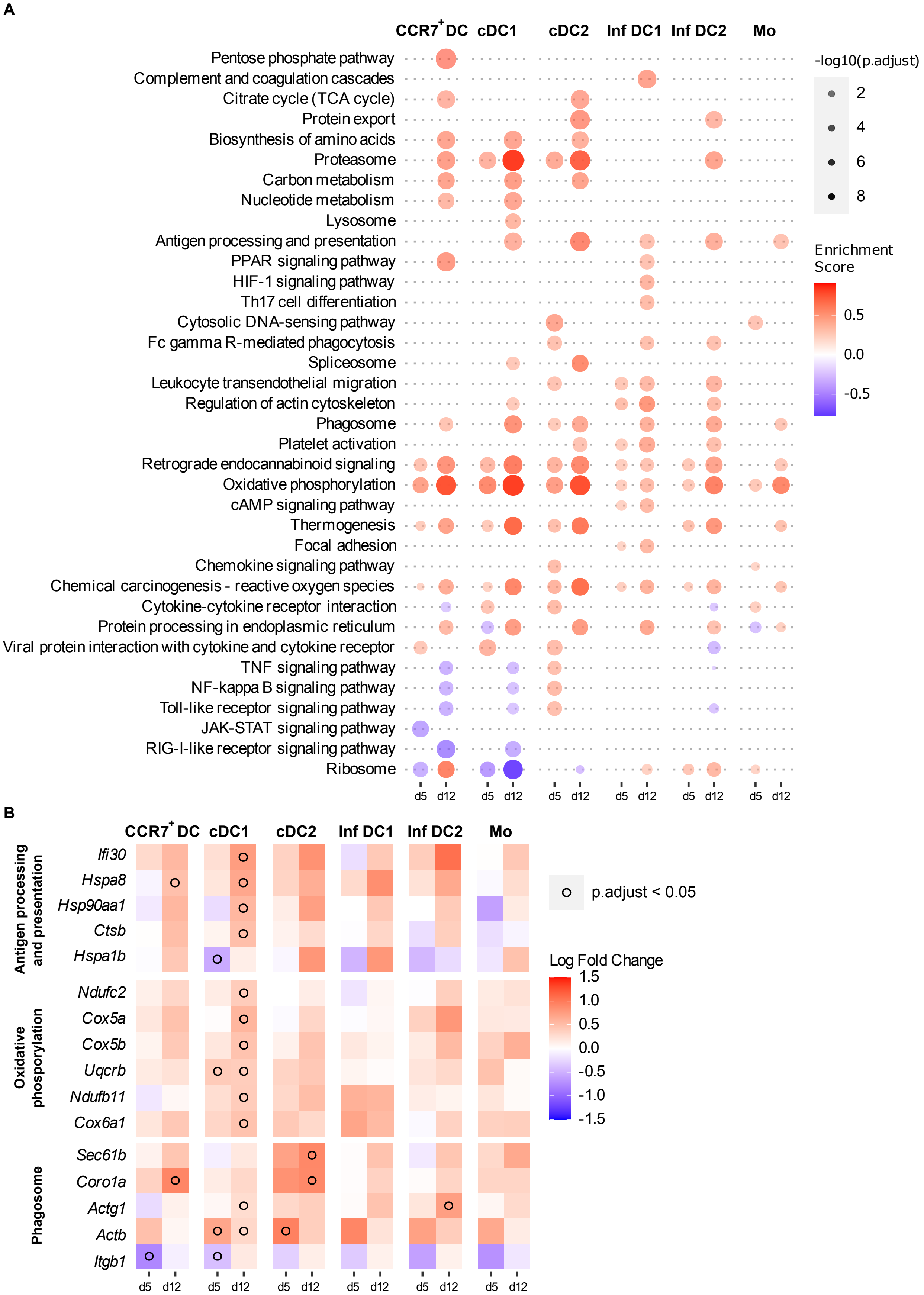

Supplementary Figure 12. Single cell RNA-sequencing reveals enrichment of metabolism-associated genes in DC subsets during fungal allergic airway inflammation. DCs were isolated from the lung tissue of naïve mice or mice that had been repeatedly exposed to *Af* spores (4 x 10^5 per dose, intranasally), harvested 24h after the third or sixth dose (*Af* d5 and *Af* d12, respectively). Single cell libraries were generated from these populations using the 10x chromium platform and sequenced (Illumina Hi-seq). 11 clusters identified by dynamic tree cluster method

were refined to 7 populations using highly expressed genes. (**A**) Dot plot representing the results of gene-set enrichment analysis. Points represent significant enrichment of KEGG pathways at *Af* d5 and *Af* d12 relative to the naive control. Point size and colour represent the enrichment score. Point opacity represents statistical significance presented as $-\log_{10}$(p.adjust). Pathways were considered significantly differentially enriched with p.adjust < 0.01 & absolute enrichment score > 0.5. The top 40 paths by absolute enrichment score are plotted. (**B**) Heat map representing the results of differential expression analysis comparing d5 and d12 post *Af* exposure to the PBS control in DC subsets. Colour represents $\log_2$(Fold Change). Circles represent where p.adjust < 0.05. Genes were selected from KEGG pathways identified as significantly enriched during anti-fungal allergic airway inflammation.

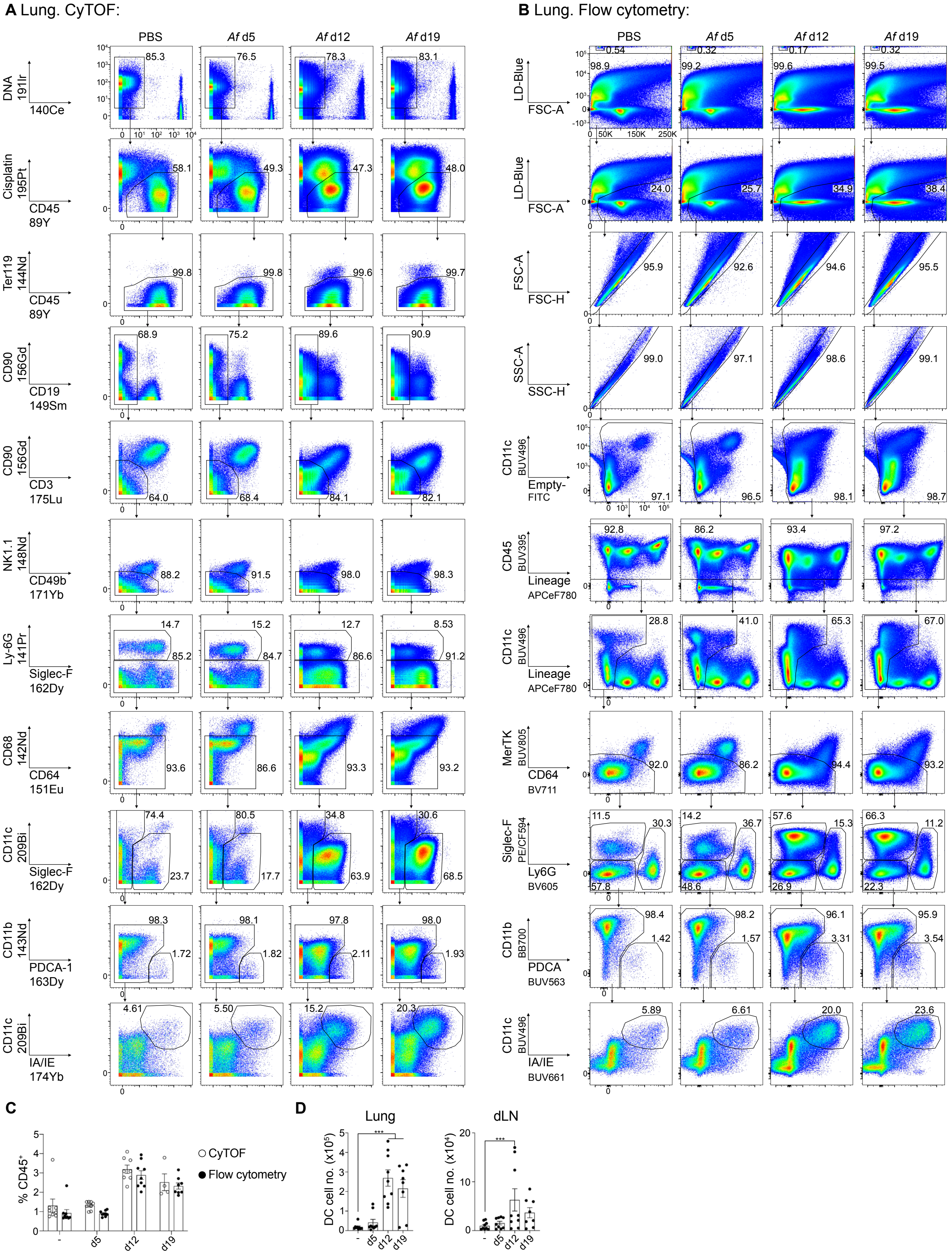

Supplementary Figure 13. Gating strategy for the identification of lung DCs via CyTOF and flow cytometry. Mice were repeatedly exposed intranasally to PBS or *Af* spores (4 x 10^5 per dose) and lung tissue was harvested the day after the third, sixth or ninth dose of spores (d5, d12 and d19, respectively). (**A**) Representative flow cytometry plots show the strategies utilised for (**A**) mass cytometry and (**B**) flow cytometry on cells isolated from the lung of PBS or *Af*-exposed mice. (**C**) Graph show the proportion of DCs as detected by CyTOF vs flow cytometry. (**D**) Graphs show the number of DCs from lung tissue as determined by flow cytometry. **C (CyTOF)**, data from 2 independent experiments (n = 28 biologically independent animals). **C (Flow cytometry) & D**, data

from 2 independent experiments (n = 35 biologically independent animals). Data were fit to a linear mixed effect model, with experimental day as a random effect variable, and groups compared with a two-sided Tukey's multiple comparison test. *p < 0.05, **p < 0.01, ***p < 0.001. Data are presented as mean values ± SEM. Source data are provided as a Source Data File.

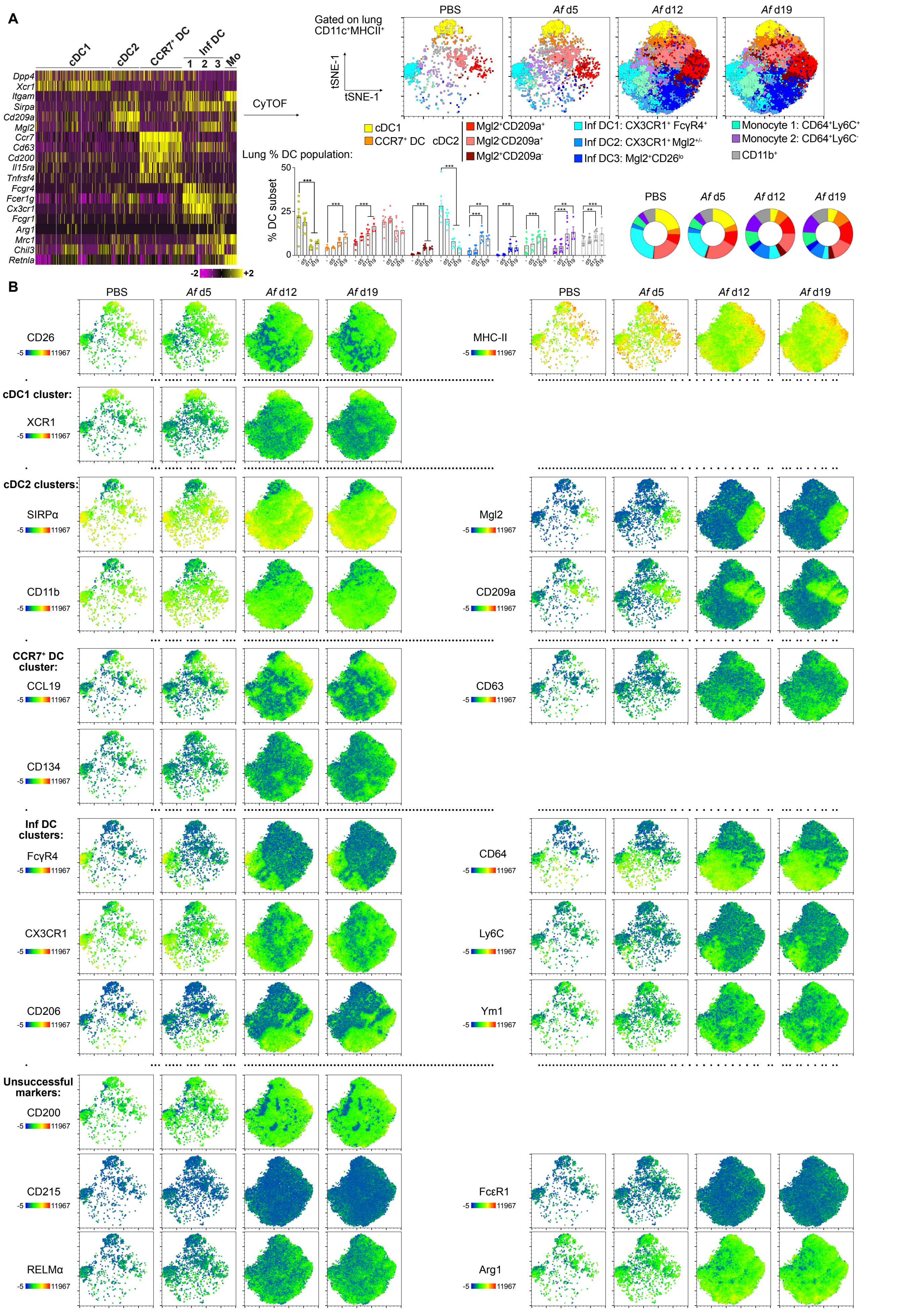
A
cDC1
cDC2
CCR7+ DC
Inf DC
1 2 3
Mo
Dpp4
Xcr1
Itgam
Sirpa
Cd209a
Mgl2
Ccr7
Cd63
Cd200
Il15ra
Tnfrsf4
Fcgr4
Fcer1g
Cx3cr1
Fcgr1
Arg1
Mrc1
Chil3
Retnla
-2
+2
CyTOF
Gated on lung CD11c+MHCII+
tSNE-1
tSNE-1
PBS
Af d5
Af d12
Af d19
cDC1
CCR7+ DC
cDC2
Mgl2+CD209a+
Mgl2-CD209a+
Mgl2+CD209a-
Inf DC1: CX3CR1+ FcγR4+
Inf DC2: CX3CR1+ Mgl2+/-
Inf DC3: Mgl2+CD26lo
Monocyte 1: CD64+Ly6C+
Monocyte 2: CD64+Ly6C-
CD11b+
Lung % DC population:
% DC subset
50
25
0
B
CD26
MHC-II
cDC1 cluster:
XCR1
cDC2 clusters:
SIRPα
Mgl2
CD11b
CD209a
CCR7+ DC cluster:
CCL19
CD63
CD134
Inf DC clusters:
FcγR4
CD64
CX3CR1
Ly6C
CD206
Ym1
Unsuccessful markers:
CD200
CD215
FcεR1
RELMα
Arg1
-5
11967

Supplementary Figure 14. Mass cytometry demonstrates that fungal allergic airway inflammation increases the proportion of cDC2 and Inf DC subsets in the lung. Mice were repeatedly exposed intranasally to PBS or *Af* spores (4 x 10^5 per dose) and lung tissue was harvested the day after the third, sixth or ninth dose of spores (d5, d12 and d19, respectively). (**A**) Identification of DC clusters in the lung tissue was achieved by designing and staining for 34 parameters via mass cytometry, analysis of DC populations utilise cluster defining hits revealed by the scRNAseq dataset. Representative tSNE plots reveal DC clusters that were identified by unbiased clustering analysis from the lung tissue of PBS or *Af*-exposed mice. Graphs and pie charts represent the proportion of each cluster within the DC population. (**B**) Representative tSNE plots reveal expression of each individual marker utilised in the clustering analysis. Unsuccessful markers, which showed minimal staining or uniform staining were excluded from the clustering analysis. Scale bars reflects staining intensity. **A**, data from 2 independent experiments (n = 28 biologically independent animals). Data were fit to a linear mixed effect model, with experimental day as a random effect variable, and groups compared with a two-sided Tukey's multiple comparison test. *p < 0.05, **p < 0.01, ***p < 0.001. Data are presented as mean values ± SEM. Source data are provided as a Source Data File.

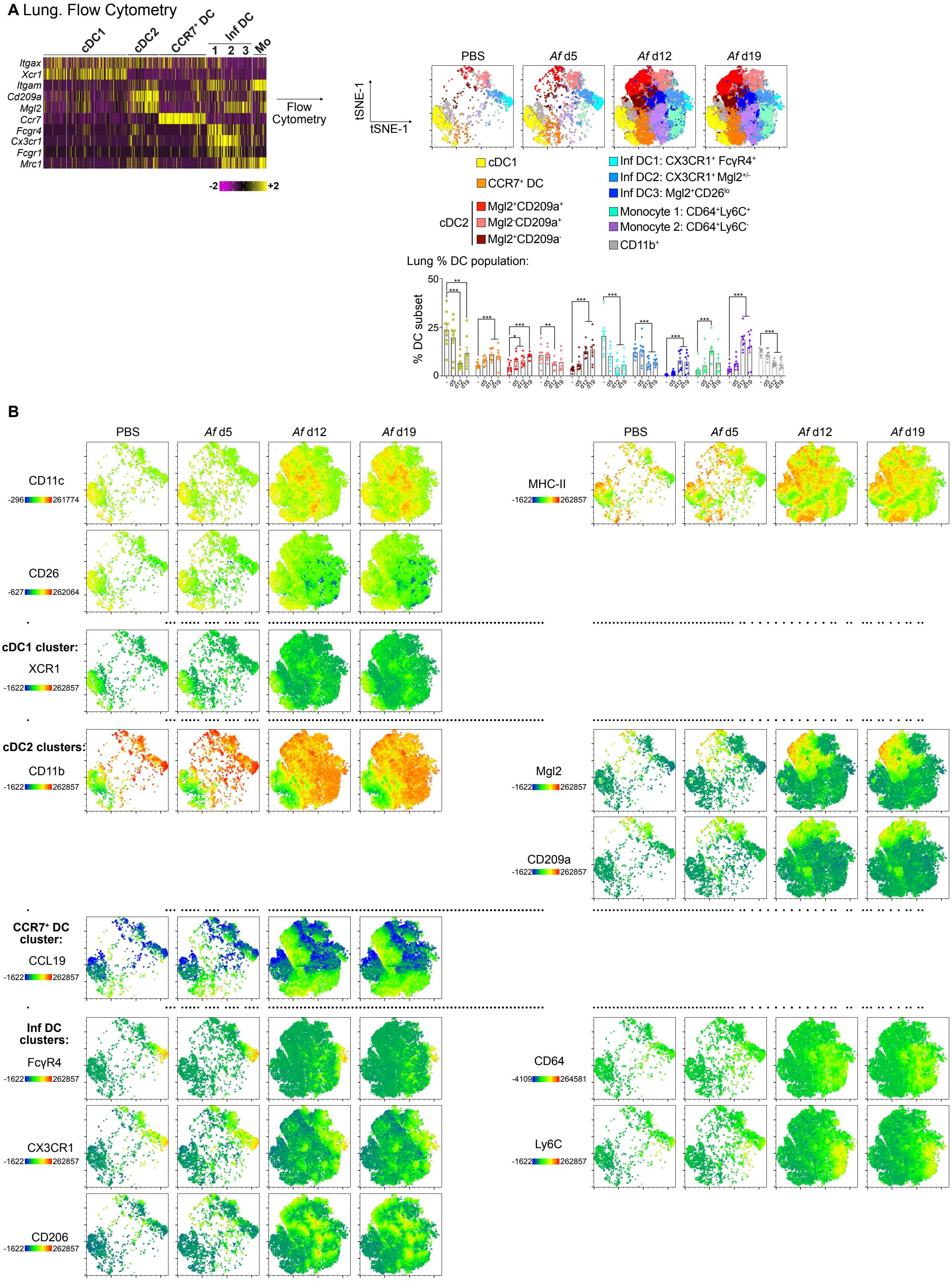

Supplementary Figure 15. Unbiased clustering analysis and identification of DC subsets in the lung tissue during fungal allergic airway inflammation revealed by flow cytometry. Mice were repeatedly exposed intranasally to PBS or *Af* spores (4 x 10^5 per dose) and lung tissue was harvested the day after the third, sixth or ninth dose of spores (d5, d12 and d19, respectively). (**A**) Identification of DC clusters in the lung tissue was achieved by designing and staining for 20 parameters via flow cytometry, analysis of DC populations utilise cluster defining hits revealed by the scRNAseq dataset. Representative tSNE plots reveal DC clusters that were identified by

unbiased clustering analysis from the lung tissue of PBS or *Af*-exposed mice. Graphs display the numbers and percentage of DC subsets. (**B**) Representative tSNE plots reveal expression of each individual marker that were included in the clustering analysis. Scale bars reflect staining intensity. **A**, data from 2 independent experiments (n = 35 biologically independent animals). Data were fit to a linear mixed effect model, with experimental day as a random effect variable, and groups compared with a two-sided Tukey's multiple comparison test. *p < 0.05, **p < 0.01, ***p < 0.001. Data are presented as mean values ± SEM. Source data are provided as a Source Data File.

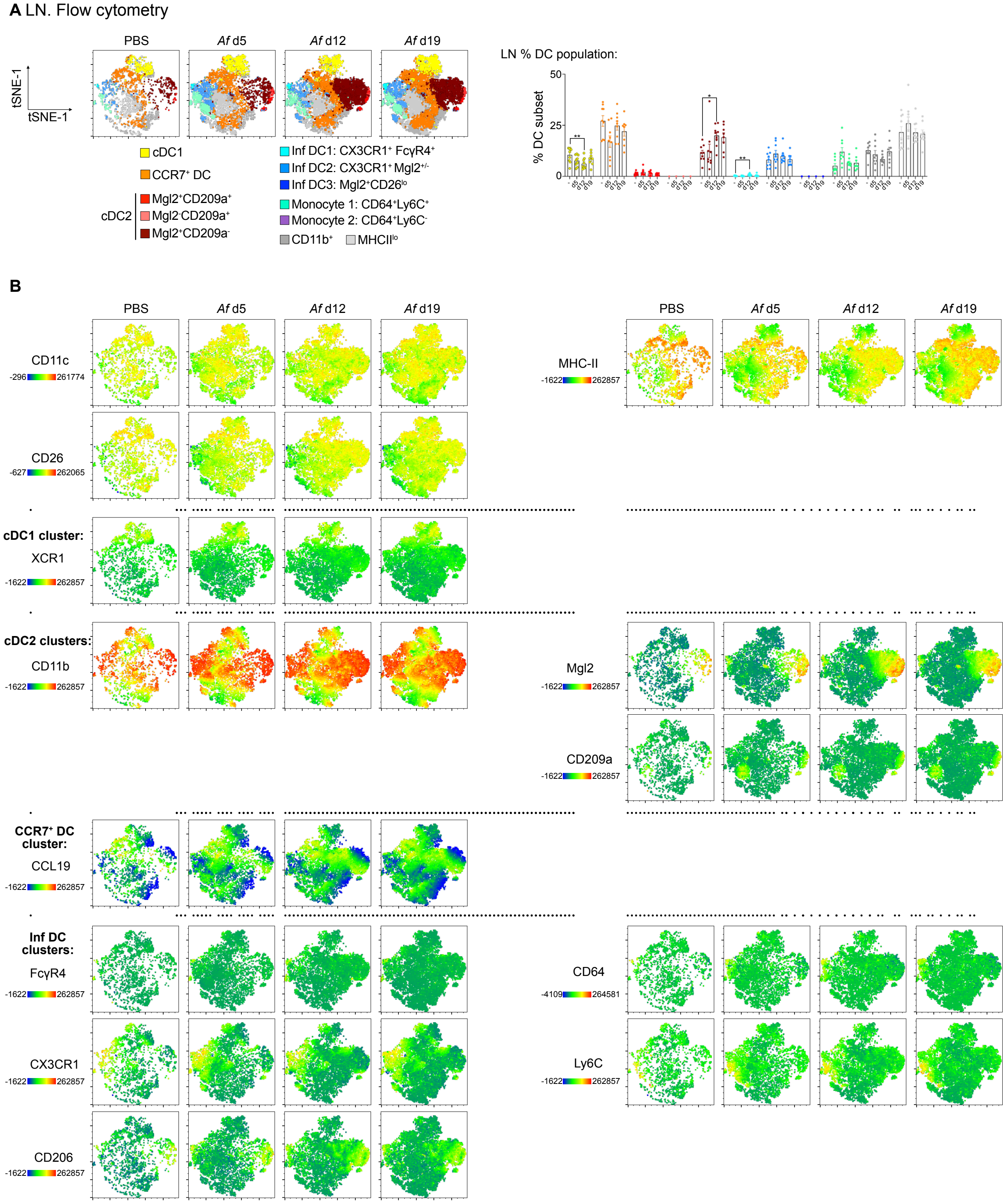

Supplementary Figure 16. Unbiased clustering analysis and identification of DC subsets in dLN revealed by flow cytometry during fungal allergic airway inflammation. Mice were repeatedly exposed intranasally to PBS or *Af* spores (4 x 10^5 per dose) and dLN tissue was harvested the day after the third, sixth or ninth dose of spores (d5, d12 and d19, respectively). (**A**) Representative tSNE plots reveal DC clusters that were identified by unbiased clustering analysis from the dLNs of PBS or *Af*-exposed mice. Graphs display the numbers and percentage of DC subsets. (**B**) Representative tSNE plots reveal expression of each individual marker utilised in the clustering analysis. Scale bars reflect staining intensity. **A**, data from 2 independent experiments (n = 35 biologically independent animals). Data were fit to a linear mixed effect model, with experimental day as a random effect variable, and groups compared with a two-sided Tukey's multiple comparison test. *p < 0.05, **p < 0.01, ***p < 0.001. Data are presented as mean values ± SEM. Source data are provided as a Source Data File.

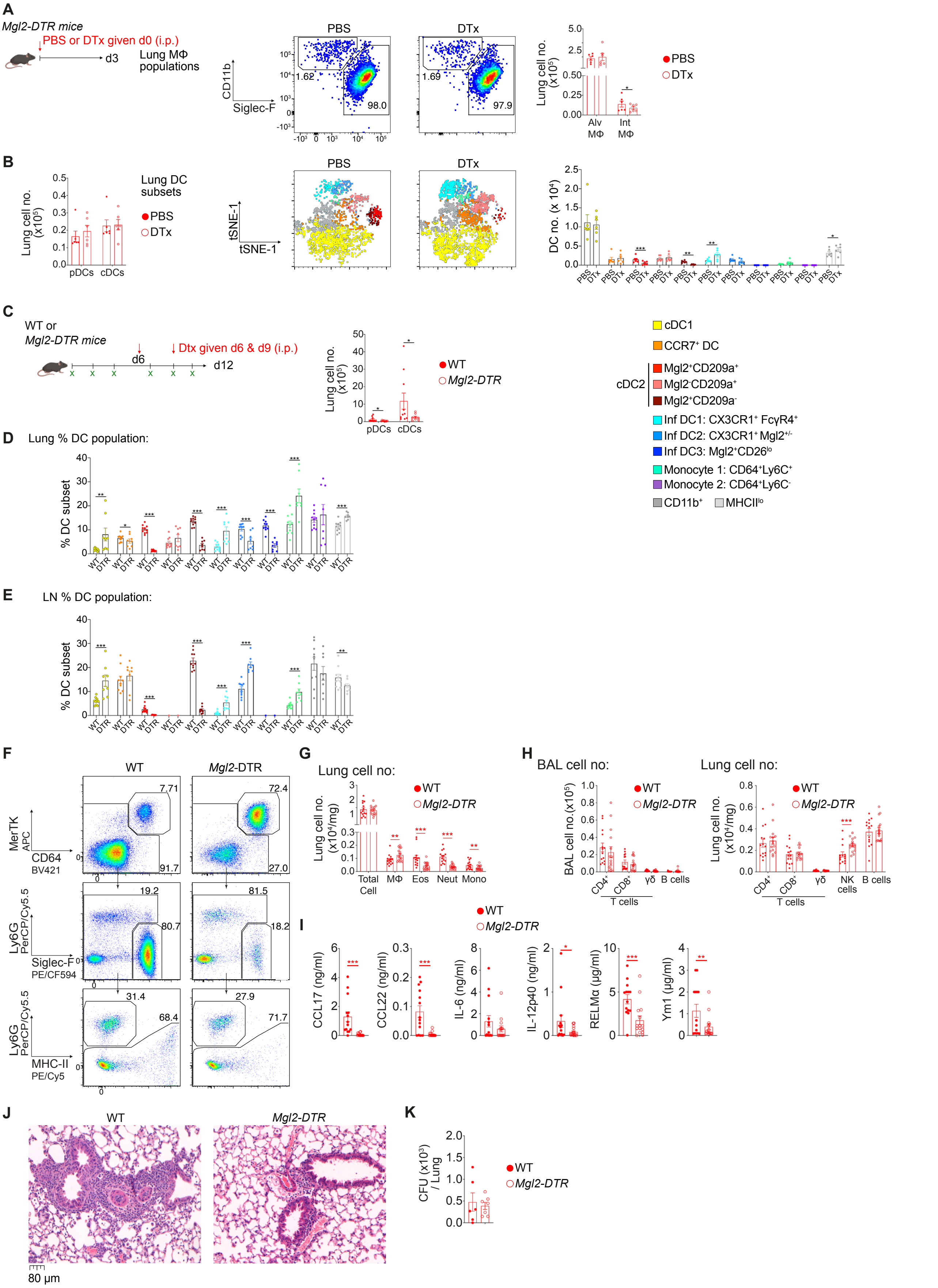

Supplementary Figure 17. Mgl2⁺ cell depletion impacts fungal allergic airway inflammation. (**A**) Naive *Mgl2*-DTR mice were treated with PBS or diphtheria toxin (DTx) and lung tissue harvested 72h later. Created in BioRender. Cook, P. (2024) https://BioRender.com/z24b189. Representative flow cytometry plots identifying macrophage (MΦ) subsets and graphs show the number of MΦs in lung tissue. (**B**) Representative flow cytometry tSNE plots show unbiased clustering analysis of

identified subsets within total DCs that were detected in lung tissue. Graphs show the number of DCs (cDCs and pDCs) or cDC subsets in lung tissue. (**C**) WT or *Mgl2*-DTR mice were treated with diphtheria toxin (DTx) (on d6 and d9) whilst being exposed to repeat doses of *Af* spores (4 x 10^5 per dose) and lung tissue was harvested 24h after the sixth dose. Created in BioRender. Cook, P. (2024) https://BioRender.com/z24b189. Graphs show the number of cDCs and pDCs in the lung tissue. (D & E) Graphs show the percentage of DC subsets in (D) lung tissue and (E) dLNs. (**F**) Representative flow cytometry plots of shows the identification of different populations in the BAL fluid. (**G**) Graph show the number of MΦ, eosinophils (Eos) neutrophils (Neut), and monocytes (Mono) from the lung tissue (**H**) Graphs show the number of lymphocyte populations from the BAL fluid and lung tissue. (**I**) ELISA of mediators detected from the BAL fluid. (**J**) Representative images of lung tissue stained with H&E. (**K**) Graph displays lung tissue fungal burden, as measured by colony forming units (CFU) of whole lung tissue. **A & B**, data from 2 independent experiments (n = 12 biologically independent animals). **C, D & E**, data from 2 independent experiments (n = 18 biologically independent animals). **G**, **H** & **I**, data from 3 independent experiments (n = 29 biologically independent animals). **K**, data from 1 experiment (n = 13 biologically independent animals). Data were fit to a linear mixed effect model, with experimental day as a random effect variable, and groups compared with a two-sided Tukey's multiple comparison test. *p < 0.05, **p < 0.01, ***p < 0.001. Data are presented as mean values ± SEM. Source data are provided as a Source Data File. Schematics in figures were created in https://BioRender.com.

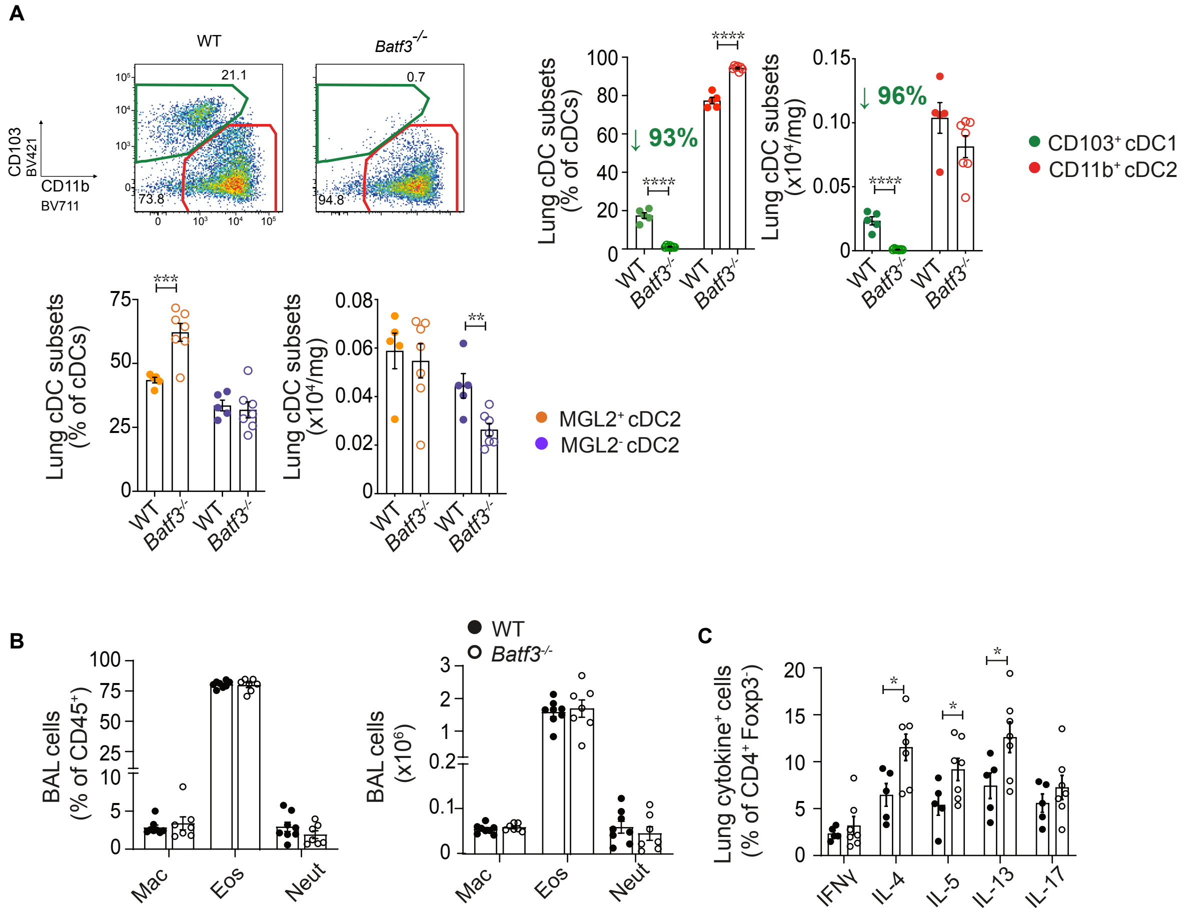

Supplementary Figure 18. Type 2 and type 17 fungal allergic airway inflammation is not dependent on *Batf3* dependent cDC1s. WT or *Batf3*$^{-/-}$ mice were repeatedly exposed intranasally to PBS or *Af* spores (4 x 10^5 per dose) and tissues were harvested the day after the ninth dose of spores (d19). (**A**) Representative flow cytometry plots identify DC populations alongside graphs that display cell numbers that were detected in the lung tissue. (**B**) Graphs show percentage and number of macrophages (MΦs), eosinophil (Eos), neutrophil (Neut) from the BAL fluid. (**C**) Graphs show percentage and number of type 2 and type 17 lung CD4$^+$ T cells. **A**, data from 2 independent experiments (n = 12 biologically independent animals). **B**, data from 2 independent experiments (n = 15 biologically independent animals). **C**, data from 2 independent experiments (n = 12 biologically independent animals). Data were fit to a linear mixed effect model, with experimental day as a random effect variable, and groups compared with a two-sided Tukey's multiple comparison test. *p < 0.05, **p < 0.01, ***p < 0.001. Data are presented as mean values ± SEM. Source data are provided as a Source Data File.
