# Supplementary material for: Mgl2+ cDC2s coordinate fungal allergic airway type 2, but not type 17, inflammation in mice
Source: Nat Commun. 2025 Jan 22;16:928. doi: 10.1038/s41467-024-55663-3 (PMC11754877; doi:10.1038/s41467-024-55663-3)
Supplement: Supplementary file 2 — Description of Additional Supplementary Files [file 41467_2024_55663_MOESM2_ESM.pdf]

Description of Additional Supplementary Files

Supplementary Data 1: Details of antibody used in study
