# Supplementary material for: Mgl2+ cDC2s coordinate fungal allergic airway type 2, but not type 17, inflammation in mice
Source: Nat Commun. 2025 Jan 22;16:928. doi: 10.1038/s41467-024-55663-3 (PMC11754877; doi:10.1038/s41467-024-55663-3)
Supplement: Supplementary file 4 — Reporting Summary [file 41467_2024_55663_MOESM4_ESM.pdf]

nature portfolio

Corresponding author(s): Peter C. Cook and Andrew S. MacDonald

Last updated by author(s): Dec 2, 2024

Reporting Summary

Nature Portfolio wishes to improve the reproducibility of the work that we publish. This form provides structure for consistency and transparency in reporting. For further information on Nature Portfolio policies, see our Editorial Policies and the Editorial Policy Checklist.

Statistics

For all statistical analyses, confirm that the following items are present in the figure legend, table legend, main text, or Methods section.

n/a	Confirmed	
☐	☒	The exact sample size (*n*) for each experimental group/condition, given as a discrete number and unit of measurement
☐	☒	A statement on whether measurements were taken from distinct samples or whether the same sample was measured repeatedly
☐	☒	The statistical test(s) used AND whether they are one- or two-sided<br>*Only common tests should be described solely by name; describe more complex techniques in the Methods section.*
☒	☐	A description of all covariates tested
☐	☒	A description of any assumptions or corrections, such as tests of normality and adjustment for multiple comparisons
☐	☒	A full description of the statistical parameters including central tendency (e.g. means) or other basic estimates (e.g. regression coefficient) AND variation (e.g. standard deviation) or associated estimates of uncertainty (e.g. confidence intervals)
☐	☒	For null hypothesis testing, the test statistic (e.g. *F*, *t*, *r*) with confidence intervals, effect sizes, degrees of freedom and *P* value noted<br>*Give P values as exact values whenever suitable.*
☒	☐	For Bayesian analysis, information on the choice of priors and Markov chain Monte Carlo settings
☒	☐	For hierarchical and complex designs, identification of the appropriate level for tests and full reporting of outcomes
☒	☐	Estimates of effect sizes (e.g. Cohen's *d*, Pearson's *r*), indicating how they were calculated

Our web collection on statistics for biologists contains articles on many of the points above.

Software and code

Policy information about availability of computer code

Data collection	BD FACS Fortessa and Symphony for flow cytometry. Helios for mass cytometry. Panoramic250 slide scanner to obtain images of histology slides. Quantstudio Pro 7 for qPCR. Illumina HiSeq4000 for scRNAseq.
Data analysis	FlowJo (v10) for flow and mass cytometry data. R and cell ranger with published scripts to analyse scRNAseq FASTQ sequence files. GraphPad Prism 10, R Studio and R for statistical analysis.

For manuscripts utilizing custom algorithms or software that are central to the research but not yet described in published literature, software must be made available to editors and reviewers. We strongly encourage code deposition in a community repository (e.g. GitHub). See the Nature Portfolio guidelines for submitting code & software for further information.

Data

Policy information about availability of data

All manuscripts must include a data availability statement. This statement should provide the following information, where applicable:

- Accession codes, unique identifiers, or web links for publicly available datasets
- A description of any restrictions on data availability
- For clinical datasets or third party data, please ensure that the statement adheres to our policy

Source data underlying Figures and Supplementary Figures are provided with this paper in the Source Data File. The scRNA-seq data has been posted on the ArrayExpress database (accession: E-MTAB-13740).

Research involving human participants, their data, or biological material

Policy information about studies with human participants or human data. See also policy information about sex, gender (identity/presentation), and sexual orientation and race, ethnicity and racism.

Reporting on sex and gender	N/A
Reporting on race, ethnicity, or other socially relevant groupings	N/A
Population characteristics	N/A
Recruitment	N/A
Ethics oversight	N/A

Note that full information on the approval of the study protocol must also be provided in the manuscript.

Field-specific reporting

Please select the one below that is the best fit for your research. If you are not sure, read the appropriate sections before making your selection.

☒ Life sciences ☐ Behavioural & social sciences ☐ Ecological, evolutionary & environmental sciences

For a reference copy of the document with all sections, see nature.com/documents/nr-reporting-summary-flat.pdf

Life sciences study design

All studies must disclose on these points even when the disclosure is negative.

Sample size	For all animal work between 4 and 21 mice were chosen per group. This sample size comes from at least two independent experiments (apart from experiment in Supplementary Fig 17K). This sample size was chosen to be sufficient based upon initial experiments with this model, taking into account the variation in the system and the resultant power of the analyses to detect the primary outcomes of each experiment. Multiple experiments with a small sample size were chosen to account for variation on the days of fungal exposure. Both male and female mice were used in experiments.
Data exclusions	For Trcd-/- experiments, 2 individual Tcrd-/- mice were removed due to disrupted lymphocyte development. When performing intracellular cytokine staining of lung cells, 3 samples were excluded when very low cell viability was recovered (<1% live) which occured in CD11c.DOG experiments.. For analysis of lung and dLN DC subsets in Mgl2-DTR experiments, 2 mice from WT group were excluded as inflammatory markers (e.g. lung eosinophila) at d12 post infection were substantially lower (likely caused with errors with with intranasal dosing) as these were not representative of wild-type responses previously observed. Two mice from Mgl2-DTRhet group, 1 due to inefficient Mgl2 cell depletion likely due to poor i.p. injection and 1 due to staining error during flow cytometry.
Replication	All experiments were performed in at least 2 independent repeats (apart from Supplementary Fig 17K), with all experiments shown in the accompanying figure. Attempts at replication were successful.
Randomization	Mice were not formally randomized. Different groups were co-housed when possible.
Blinding	Blinding was not used for this study. The study was not considered to be susceptible to the subjectivity of the researcher, with measurements and analysis techniques used applied equally to each group (flow cytometry gate positions for instance).

Reporting for specific materials, systems and methods

We require information from authors about some types of materials, experimental systems and methods used in many studies. Here, indicate whether each material, system or method listed is relevant to your study. If you are not sure if a list item applies to your research, read the appropriate section before selecting a response.

Materials & experimental systems

n/a	Involved in the study
	x Antibodies
x	Eukaryotic cell lines
x	Palaeontology and archaeology
	x Animals and other organisms
x	Clinical data
x	Dual use research of concern
x	Plants

Methods

n/a	Involved in the study
x	ChIP-seq
	x Flow cytometry
x	MRI-based neuroimaging

Antibodies

Antibodies used

Biolegend
Antigen: CD103 , Clone: 2E7 , flourochrome: BV421 , titration 1:200 , Cat no: 121422
Antigen: CD11b , Clone: M1/70 , flourochrome: BV711 , titration 1:600 , Cat no:101242
Antigen: CD11c , Clone: N418 , flourochrome: BV605 , titration 1:200 , Cat no: 117334
Antigen: CD206 , Clone: C068C2 , flourochrome: BV785 , titration 1:100 , Cat no: 141729
Antigen: CD209a , Clone: MMD3 , flourochrome: PE , titration 1:200 , Cat no: 833004
Antigen: CD25 , Clone: PC61 , flourochrome: BV605 , titration 1:100 , Cat no: 102036
Antigen: CD301b , Clone: URA-1 , flourochrome: PE , titration 1:50 , Cat no: 146804
Antigen: CD301b , Clone: URA-1 , flourochrome: PE/Cy7 , titration 1:400 , Cat no: 146808
Antigen: CD317 (PDCA1) , Clone: 927 , flourochrome: BV650 , titration 1:100 , Cat no: 127019
Antigen: CD4 , Clone: RM4-5 , flourochrome: AF700 , titration 1:200 , Cat no: 100536
Antigen: CD45 , Clone: 30-F11 , flourochrome: BV510 , titration 1:500 , Cat no:103138
Antigen: CD45 , Clone: 30-F11 , flourochrome: BV785 , titration 1:500 , Cat no:103149
Antigen: CD45R (B220) , Clone: RA3-6B2 , flourochrome: BV605 , titration 1:200 , Cat no: 103243
Antigen: CD64 , Clone: X54-5/7.1 , flourochrome: PE , titration 1:100 , Cat no: 161004
Antigen: CD64 , Clone: X54-5/7.1 , flourochrome: BV711 , titration 1:100 , Cat no: 139311
Antigen: CD8 , Clone: 53-6.7 , flourochrome: PE/Cy7 , titration 1:800 , Cat no: 100722
Antigen: CX3CR1 , Clone: SA011F11 , flourochrome: PE/Dazzle594 , titration 1:100 , Cat no: 149014
Antigen: FcγR4 , Clone: 9E9 , flourochrome: BV421 , titration 1:100 , Cat no: 149521
Antigen: IA/IE , Clone: M5/114.15.2 , flourochrome: PE/Cy5 , titration 1:1000 , Cat no: 107612
Antigen: IFNγ , Clone: XMG1.2 , flourochrome: BV711 , titration 1:200 , Cat no: 505836
Antigen: IL-10 , Clone: JES5-16E3 , flourochrome: BV605 , titration 1:200 , Cat no: 505031
Antigen: IL-17 , Clone: TC11-18H10.1 , flourochrome: PE/Cy7 , titration 1:200 , Cat no: 100202
Antigen: IL-5 , Clone: TRFK5 , flourochrome: PE , titration 1:200 , Cat no: 504304
Antigen: Ly6C , Clone: HK1.4 , flourochrome: BV605 , titration 1:100 , Cat no: 128036
Antigen: Ly6C , Clone: HK1.4 , flourochrome: AF700 , titration 1:200 , Cat no: 128024
Antigen: Ly6G , Clone: 1A8 , flourochrome: BV650 , titration 1:600 , Cat no: 127641
Antigen: Ly6G , Clone: 1A8 , flourochrome: FITC , titration 1:400 , Cat no: 127606
Antigen: Ly6G , Clone: 1A8 , flourochrome: PerCP/Cy5.5 , titration 1:400 , Cat no: 127616
Antigen: MerTK , Clone: 2B10C42 , flourochrome: FITC , titration 1:50 , Cat no: 151504
Antigen: NK1.1 , Clone: PK136 , flourochrome: PE/Cy5 , titration 1:200 , Cat no: 108716
Antigen: TCRγδ , Clone: GL3 , flourochrome: PE , titration 1:200 , Cat no: 118108
Antigen: TCRγδ , Clone: GL3 , flourochrome: PE/Cy7 , titration 1:200 , Cat no: 118124
Antigen: XCR1 , Clone: ZET , flourochrome: BV510 , titration 1:100 , Cat no: 148218
Antigen: CD19 , Clone: 1D3 , Biotin , titration 1:200 , Cat no: 152420
Antigen: CD3 , Clone: 17A2 , Biotin , titration 1:100 , Cat no: 100244
Antigen: CD45R (B220) , Clone: RA3-6B2 , Biotin , titration 1:200 , Cat no: 103204
Antigen: CD90.2 , Clone: 30-H12 , Biotin , titration 1:200 , Cat no: 105348
Antigen: Ly6G , Clone: 1A8 , Biotin , titration 1:200 , Cat no: 127604
Antigen: NK1.1 , Clone: PK136 , Biotin , titration 1:200 , Cat no: 108704
Antigen: Ter119 , Clone: TER119 , Biotin , titration 1:200 , Cat no: 116204
Antigen: CD134 , Clone: OX-86 , Purified , titration 1:50 , Cat no: 119429
Antigen: CD172a (SIRPα) , Clone: P84 , Purified , titration 1:50 , Cat no: 144002
Antigen: CD200 , Clone: OX2 , Purified , titration 1:50 , Cat no: 123802
Antigen: CD209a , Clone: MMD3 , Purified , titration 1:50 , Cat no: 833001
Antigen: CD215 , Clone: 6B4C88 , Purified , titration 1:50 , Cat no: 153502
Antigen: CD26 , Clone: H194-112 , Purified , titration 1:50 , Cat no: 137802
Antigen: CD3 , Clone: 17A2 , Purified , titration 1:50 , Cat no: 100202
Antigen: CD317 (PDCA1) , Clone: 927 , Purified , titration 1:50 , Cat no: 127004
Antigen: CD49b , Clone: DX5 , Purified , titration 1:50 , Cat no: 108902
Antigen: CD63 , Clone: NVG-2 , Purified , titration 1:50 , Cat no: 143902
Antigen: CD68 , Clone: FA-11 , Purified , titration 1:50 , Cat no: 137002

Antigen: CX3CR1 , Clone: SA011F11 , Purified , titration 1:50 , Cat no: 149011
Antigen: F4/80 , Clone: BM8 , Purified , titration 1:50 , Cat no: 123102
Antigen: FcγR4 , Clone: 9E9 , Purified , titration 1:50 , Cat no: 149502
Antigen: NK1.1 , Clone: PK136 , Purified , titration 1:50 , Cat no: 108702
Antigen: Ter119 , Clone: TER119 , Purified , titration 1:50 , Cat no: 116202
Antigen: XCR1 , Clone: ZET , Purified , titration 1:50 , Cat no: 148202

ThermoFisher
Antigen: CD19 , Clone: 1D3 , flourochrome: APCeF780 , titration 1:100 , Cat no: 47-0193-82
Antigen: CD3 , Clone: 17A2 , flourochrome: APCeF780 , titration 1:100 , Cat no: 47-0032-82
Antigen: CD90.2 , Clone: 30-H12 , flourochrome: APCeF780 , titration 1:200 , Cat no: 47-0903-82
Antigen: CD90.2 , Clone: 30-H12 , flourochrome: eF450 , titration 1:200 , Cat no: 62-0903-82
Antigen: IL-13 , Clone: eBio13A , flourochrome: AF488 , titration 1:200 , Cat no: 53-7133-82
Antigen: Ly6G , Clone: 1A8 , flourochrome: APCeF780 , titration 1:200 , Cat no: 47-9668-82
Antigen: NK1.1 , Clone: PK136 , flourochrome: APCeF780 , titration 1:200 , Cat no: 47-5941-82
Antigen: TCRb , Clone: H57-597 , flourochrome: APCeF780 , titration 1:200 , Cat no: 47-5961-82
Antigen: Ter119 , Clone: TER119 , flourochrome: APCeF780 , titration 1:200 , Cat no: 47-5921-82
Antigen: Slc7a2 , Polyclonal , Purified , titration 1:50 , Cat no: PA5-69252

Miltenyi Biotec
Antigen: Siglec-F , Clone: ES22-10D8 , flourochrome: APC , titration 1:50 , Cat no: 130-123-816

BD
Antigen: CD11b , Clone: M1/70 , flourochrome: BB700 , titration 1:5000 , Cat no: 566416
Antigen: CD11c , Clone: N418 , flourochrome: BUV496 , titration 1:100 , Cat no: 750450
Antigen: CD19 , Clone: 1D3 , flourochrome: PE/CF594 , titration 1:200 , Cat no: 562291
Antigen: CD26 , Clone: H194-112 , flourochrome: BUV737 , titration 1:100 , Cat no: 741729
Antigen: CD317 (PDCA1) , Clone: 927 , flourochrome: BUV563 , titration 1:100 , Cat no: 749275
Antigen: CD45 , Clone: 30-F11 , flourochrome: BUV395 , titration 1:500 , Cat no: 564279
Antigen: IA/IE , Clone: M5/114.15.2 , flourochrome: BUV661 , titration 1:1000 , Cat no: 750280
Antigen: IL-4 , Clone: 11B11 , flourochrome: PE/CF594 , titration 1:200 , Cat no: 562450
Antigen: Siglec-F , Clone: E50-2440 , flourochrome: APC-R700 , titration 1:100 , Cat no: 565183
Antigen: Siglec-F , Clone: E50-2440 , flourochrome: PE/CF594 , titration 1:200 , Cat no: 562757
Antigen: CD16/32 , Clone: 2.4G2 , Purified , titration 1:200 , Cat no: 553142

R&D
Antigen: MerTK , Polyclonal , Biotin , titration 1:100 , Cat no: AF591
Antigen: Arginase 1 , Polyclonal , Purified , titration 1:50 , Cat no: AF5868
Antigen: Ym1 , Polyclonal , Purified , titration 1:50 , Cat no: AF2446

Peprotech
Antigen: Retnla , Polyclonal , Purified , titration 1:50 , Cat no: 500-P214-100UG

Standard Biotools
Antigen: Anti-APC , Clone: APC003 , tag: 162Dy , titration 1:100 Cat no: 3162006B
Antigen: Anti-Biotin , Clone: 1D4-C5 , tag: 170Er , titration 1:100 Cat no: 3170003B
Antigen: Anti-FITC , Clone: FIT-22 , tag: 160Gd , titration 1:100 Cat no: 3160011B
Antigen: Anti-PE , Clone: PE001 , tag: 165Ho , titration 1:100 Cat no: 3165015B
Antigen: CD11b , Clone: M1/70 , tag: 143Nd , titration 1:100 Cat no: 3143015B
Antigen: CD11c , Clone: N418 , tag: 209Bi , titration 1:100 Cat no: 3209005B
Antigen: CD19 , Clone: 6D5 , tag: 149Sm , titration 1:100 Cat no: 3149002C
Antigen: CD206 , Clone: C068C2 , tag: 169Tm , titration 1:100 Cat no: 3169021B
Antigen: CD45 , Clone: 30-F11 , tag: 89Y , titration 1:100 Cat no: 3089005B
Antigen: CD64 , Clone: X54-5/7.1 , tag: 151Eu , titration 1:100 Cat no: 3151012B
Antigen: CD90 , Clone: 30-H12 , tag: 156Gd , titration 1:100 Cat no: 3156006B
Antigen: FcεR1 , Clone: Mar-1 , tag: 176Yb , titration 1:100 Cat no: 3176006B
Antigen: IA/IE , Clone: M5/114.15.2 , tag: 174Yb , titration 1:100 Cat no: 3174003C
Antigen: Ly6C , Clone: HK1.4 , tag: 150Nd , titration 1:100 Cat no: 3150010B
Antigen: Ly6G , Clone: 1A8 , tag: 141Pr , titration 1:100 Cat no: 3141008C

Validation

Antibodies were verified as listed on the manufacturers websites. The list above states which companies the antibodies were purchased from.

Animals and other research organisms

Policy information about studies involving animals; ARRIVE guidelines recommended for reporting animal research, and Sex and Gender in Research

Laboratory animals	This study used several transgenic mouse strains. The following strains Batf3-/- (ref 26), Bdca2-DTR (ref 46), CD11c.DOG (ref 85), MGL2-DTR (ref 43) and Tcrd-/- were bred as homozygous so wild type mice were sourced from different breeders. For the majority of the experiments, MGL2-DTR het mice were generated with Pep3 (congenic CD45.1 strain). C57BL/6 x Pep3 mice were used as WT control group. Pep3 mice and CD57BL/6 mice displayed responses to repeat doses of spores. Dual IL-13 and IL17 reporters were generated with breeding homozygous Il-13eGFP (ref35) and IL17CreROSAeYFP (ref 34) mice, resulting heterozygous offpsring were used in experiments. Mice were housed in individually ventilated cages, with cage temperature at 23 degrees C , humidity at 54% and a 12hr light/ dark cycle.
Wild animals	No wild animals were used in this study.
Reporting on sex	Findings relate to both sexes. Sex was not considered in study design Both sexes were included in all analyses.
Field-collected samples	N/A
Ethics oversight	Murine work was performed under the terms of a UK Home Office approved Project License (P44492AC9 held by A.S.M.) at the Biological Services Facility at the University of Manchester (UoM) or Project License (PP6094315 held by P.C.C.) at the Biological Services Unit at the University of Exeter (UoE). All procedures were ethically reviewed, performed under license and in accordance with the UK Home Office ASPA 1986 and the GSK Policy on the Care, Welfare and Treatment of Animals as well as the European Union Animals Directive 2010/63/EU and were approved by UoM's and UoE's Animal Welfare and Ethical Review Body (AWERB).

Note that full information on the approval of the study protocol must also be provided in the manuscript.

Plants

Seed stocks	N/A
Novel plant genotypes	N/A
Authentication	N/A

Flow Cytometry

Plots

Confirm that:

- [x] The axis labels state the marker and fluorochrome used (e.g. CD4-FITC).
- [x] The axis scales are clearly visible. Include numbers along axes only for bottom left plot of group (a 'group' is an analysis of identical markers).
- [x] All plots are contour plots with outliers or pseudocolor plots.
- [x] A numerical value for number of cells or percentage (with statistics) is provided.

Methodology

Sample preparation	Isolation of immune cells from the BAL, lung and dLN.<br>Following killing, BAL cells were obtained by washing the lung airway with PBS containing 2% FBS and 2 mM EDTA (Sigma). Lungs were processed as previously described54, briefly lung tissue was chopped and incubated at 37ºC whilst shaking for 30 min with 0.8 U/ml Liberase TL and 80 U/ml DNase in HBSS (all Sigma). In contrast, dLN (not disrupted prior to incubation) were incubated at 37ºC whilst shaking for 30 min with 1 U/ml Liberase TL and 80 U/ml DNase in HBSS (all Sigma). To stop digestions, ice cold PBS containing 2% FBS and 2 mM EDTA was added and then passed through a 70 µM cell strainer.<br><br>Lung cell stimulation, flow cytometry staining and acquisition<br>For measurement of immune cell cytokine secretion, lung single cell suspensions were incubated with PMA (30 ng/ml, Sigma), Ionomycin (1 µg/ml, Sigma) and GolgiStop (BD) for 3 hours prior to surface and then intracellular staining. For surface staining, equal numbers of cells were stained for each sample, washed with ice-cold PBS and stained with Live/Dead Blue (ThermoFisher) or Zombie UV dye (BioLegend) for 10 min at room temperature. Samples were then incubated with αCD16/CD32 (2.4G2; BD Biosciences) in FACS buffer (PBS containing 2% FBS and 2 mM EDTA) before staining for surface markers at 4˚C for 60 min (antibodies and labelled chemokines listed in Supplementary Table 2). Post staining, cells were washed twice

in FACS buffer and then fixed in 1% paraformaldehyde in PBS for 10 min at room temperature. For detection of intracellular antigens, cells were processed with Foxp3 buffer staining set (ThermoFisher) and stained with selected antibodies. Samples were acquired on a BD Fortessa or BD Symphony with FacsDiva (BD) software. For most experiments, we counted total cells recovered from BAL fluid or lung tissue digestions using a hemocytometer and trypan blue, to exclude dead cells, prior to staining for flow cytometry. In some experiments CountBright Absolute Counting Beads (ThermoFisher) were added to samples prior to flow cytometry acquisition to calculate absolute cell counts.

CyTOF staining

Samples were stained following the flow cytometry staining protocol. Cells were washed in PBS before incubation with 1.25 nM Cisplatin solution prior to addition of αCD16/CD32 (2.4G2; BD) in FACS buffer (PBS containing 2% FBS and 2 mM EDTA) before staining for two rounds of surface markers at 4˚C for 60 minutes (antibodies and labelled chemokines listed in Supplementary Table 2). Post staining, cells were washed twice in FACS buffer and then fixed in 1% paraformaldehyde in PBS for 10 min at room temperature. For detection of intracellular antigens, cells were fixed with Foxp3 buffer staining set (ThermoFisher) and stained with selected antibodies. Once stained samples were frozen in 10% DMSO (Sigma) 50% FBS and RPMI prior to being run on the CyTOF Helios. Frozen samples were thawed on ice and washed once in MAXPAR PBS, and twice in milliQ water before being resuspended in MilliQ water at 0.5 x 10^6 cells / mL. EQ beads for post-acquisition normalization were added to the sample and data was acquired on a Helios mass cytometer (Standard Biotools). Resultant *.FCS files were normalized with the CyTOFv2 software before downstream analysis.

Instrument	BD Fortessa, BD Symphony and Standard Biotools Helios mass cytometer
Software	Flow Cytometry instruments: BD; FACSDiva, CYTOF (Standard Biotools). Analysis via FlowJo (v10).
Cell population abundance	Lung samples from naïve or Af-exposed mice were digested as mentioned above and OptiPrep (Stemcell Technologies) gradient was used to enrich DCs. Briefly, lung single cell suspension was resuspended in 15% OptiPrep then 11.5% OptiPrep followed by HBSS was layered on top to form distinct layers. Gradients were spun at 600g for 15 min with no brake and a band of mononuclear cells at the top of the gradient was collected. DCs were then isolated via flow sorting (CD3-CD19-CD45R-Ly6G-MerTK-NK1.1-Siglec-F-Ter119-, CD45+CD11c+IA/IE+) using an Influx (BD BioSciences) to a purity of 95-99%. Small fraction of each purified population was rerun to confirm purity.
Gating strategy	Gating strategies are shown in supplementary figures.

☒ Tick this box to confirm that a figure exemplifying the gating strategy is provided in the Supplementary Information.
